# Cyclic AMP is a global virulence regulator governing inter and intrabacterial signalling in *Acinetobacter baumannii*

**DOI:** 10.1371/journal.ppat.1012529

**Published:** 2024-09-06

**Authors:** Lyuboslava G. Harkova, Rubén de Dios, Alejandro Rubio-Valle, Antonio J. Pérez-Pulido, Ronan R. McCarthy

**Affiliations:** 1 Antimicrobial Innovations Centre, Division of Biosciences, Department of Life Sciences, College of Health and Life Sciences, Brunel University London, Uxbridge, United Kingdom; 2 Centro Andaluz de Biología del Desarrollo (CABD-CSIC-JA), Universidad Pablo de Olavide, Sevilla, Spain; Washington University in Saint Louis School of Medicine, UNITED STATES OF AMERICA

## Abstract

*Acinetobacter baumannii* is an opportunistic nosocomial pathogen with high morbidity and mortality rates. Current treatment options for this pathogen are limited due to its increasing resistance to last-resort antibiotics. Despite *A*. *baumannii’s* leading position in the World Health Organisations priority pathogens list, little is known about its virulence regulation. Through a high-throughput screening approach to identify novel biofilm regulators, we identified a previously uncharacterised predicted adenylate cyclase (AC), CavA, as a central regulator of this phenotype. cAMP is a crucial mediator of various aspects of bacterial physiology in other species but information about its role in *A*. *baumannii* is limited. We confirm that CavA AC is functional and synthesizes cAMP in *A*. *baumannii*. Using dRNA-seq, we verify that CavA is a negative biofilm formation regulator affecting Csu pili and exopolysaccharide production. We demonstrate for the first time that in *A*. *baumannii*, cAMP is atop of a hierarchical signalling cascade controlling inter- and intrabacterial signalling by modulating quorum sensing and cyclic di-GMP systems, ultimately governing virulence *in vivo* and adaptive antibiotic resistance. In contrast to the well-established paradigm in other bacteria where cAMP and cyclic di-GMP levels are inversely regulated, we uncover that the levels of these second messengers are directly proportional in *A*. *baumannii*. Overall, this study uncovers the central role of CavA and cAMP in the pathogenic success of *A*. *baumannii* and highlights this signalling cascade as a high potential target for novel therapeutic development.

## Author summary

*Acinetobacter baumannii* is at the top of the World Health Organisation’s list of critical pathogens in urgent need of novel therapeutic interventions as currently treatments options routinely fail due to its high resistance to antibiotics. Despite the importance of this pathogen, we have limited knowledge of how *A*. *baumannii* regulates virulence or antimicrobial susceptibility. In this study we show that CavA, an enzyme responsible for the production of the second messenger molecule cyclic AMP (cAMP), decreases biofilm formation while making the bacterium more motile. We demonstrate for the first time in this pathogen, that cAMP controls other signalling systems such as quorum sensing. Also, cAMP increases the levels of another important second messenger molecule cyclic di-GMP, which does not follow the current dogma established in other bacteria. Finally, we show that cAMP increases *A*. *baumannii* antibiotic resistance and virulence and provide evidence that cAMP controls many of these phenotypes by binding to the cAMP effector protein Vfr. Altogether, this study presents the important role of cAMP signalling in the success of *A*. *baumannii* as human pathogen. This will help develop new therapeutics for better treatment of infections caused by this critical-priority bacterium.

## Introduction

Antimicrobial resistance (AMR) poses one of the main global health challenges of the 21^st^ century with estimated nearly 5 million annual deaths associated with AMR and 1.27 million deaths due to AMR [1]. *A*. *baumannii* is one of the main pathogens responsible for 250,000 AMR-related and 132,000 AMR-attributed deaths annually [1]. *A*. *baumannii* opportunistically infects immunocompromised people causing a range of community-acquired and nosocomial infections [2,3]. Of particular concern is its prevalence in hospital settings [4]. Most of the clinical isolates are multidrug resistant (MDR) and the emergence and prevalence of Carbapenem-resistant isolates is a major concern globally [4].

While much is known about the varied arsenal of resistance mechanisms to both antibiotics and disinfectants encoded by *A*. *baumannii*, comparatively little is known about this pathogen’s regulation of virulence [5–7]. *A*. *baumannii* has a diverse range of virulence factors contributing to its pathogenicity such as motility, attachment and biofilm formation. Biofilms are multifaceted bacterial communities that provide protection from challenges such as the host immune system and antimicrobials. Formation and maintenance of mature biofilms requires a number of factors including various proteins and pili as well as exopolysaccharides (EPS), which are an essential part of the extracellular matrix [8]. Apart from being important for biofilm formation, pili mediate various aspects of *A*. *baumannii* lifestyle. Separate gene clusters encode the components of different classes of pili with distinct functions. Type IV pili, encoded by different *pil* genes, are crucial for *A*. *baumannii* twitching motility and natural transformation [9–11]. On the other hand, the *csuA/BABCDE* operon encodes chaperone-usher pili needed for attachment to hydrophobic surfaces and host epithelial cells, biofilm formation and virulence [12].

*A*. *baumannii* virulence is mediated by various signalling cascades ranging from single- and two-component systems (TCS) to intra- and interspecies signalling. The transition to the biofilm lifestyle, which is a major virulence determinant, is dependent on population density, monitored via the secretion of autoinducing molecules. Autoinducers (AIs) are part of the quorum sensing (QS) system and their concentration is proportional to the number of the bacterial population. *A*. *baumannii* has a single LuxI/LuxR type QS system for intercellular signalling. It consists of the autoinducer synthase AbaI producing N-(3-hydroxydodecanoyl)-L-homoserine lactone (3-OH-C_12_-HSL) which effector protein is the receptor AbaR, as well as the negative regulator of the system AbaM [13–15]. QS controls the expression of different genes implicated in motility, attachment, biofilm formation, virulence and antibiotic resistance [14,16,17]. Other important regulatory systems are the intracellular second messenger signalling cascades. However, compared to other high priority pathogens, comparatively little is known about the role of second messenger signalling cascades in *A*. *baumannii*, although there is an indication that second messengers may play an important role in *A*. *baumannii* physiology, with for example a homolog of the known phosphodiesterase CpdA having a role in pellicle formation [18]. Two studies have previously linked c-di-GMP to biofilm formation and motility in the lab adapted strain *A*. *baumannii* ATCC17978 [19,20]. An established paradigm, based on studies in other bacteria, is the antagonistic work of these two major second messengers [21–23]. However, particularly within the context of MDR clinical isolates, the role of second messengers and the interplay between the signalling systems in *A*. *baumannii* is not clear.

In this work we uncover the central role of cAMP in the regulation of pathogenicity and virulence factors in a multidrug resistant (MDR) clinical isolate of *A*. *baumannii*, AB5075. We demonstrate that the previously uncharacterised CavA (*ABUW_2208*) is the only functional adenylate cyclase in AB5075. We then use a clean deletion mutant of *cavA* to uncover the central role cAMP plays in regulating many of the phenotypes that contribute to the pathogenic success of *A*. *baumannii*. We demonstrate for the first time that cAMP sits at the top of a hierarchical signalling cascade controlling both QS and c-di-GMP signalling systems in *A*. *baumannii*. In contrast to the current paradigm, we demonstrate that the two second messengers are directly proportional in *A*. *baumannii*. Furthermore, our data demonstrates the role of cAMP as a global regulator of pathogenicity in *A*. *baumannii*.

## Results

### *ABUW_2208* is a negative regulator of *A*. *baumannii* biofilm formation

**Fig 1:**
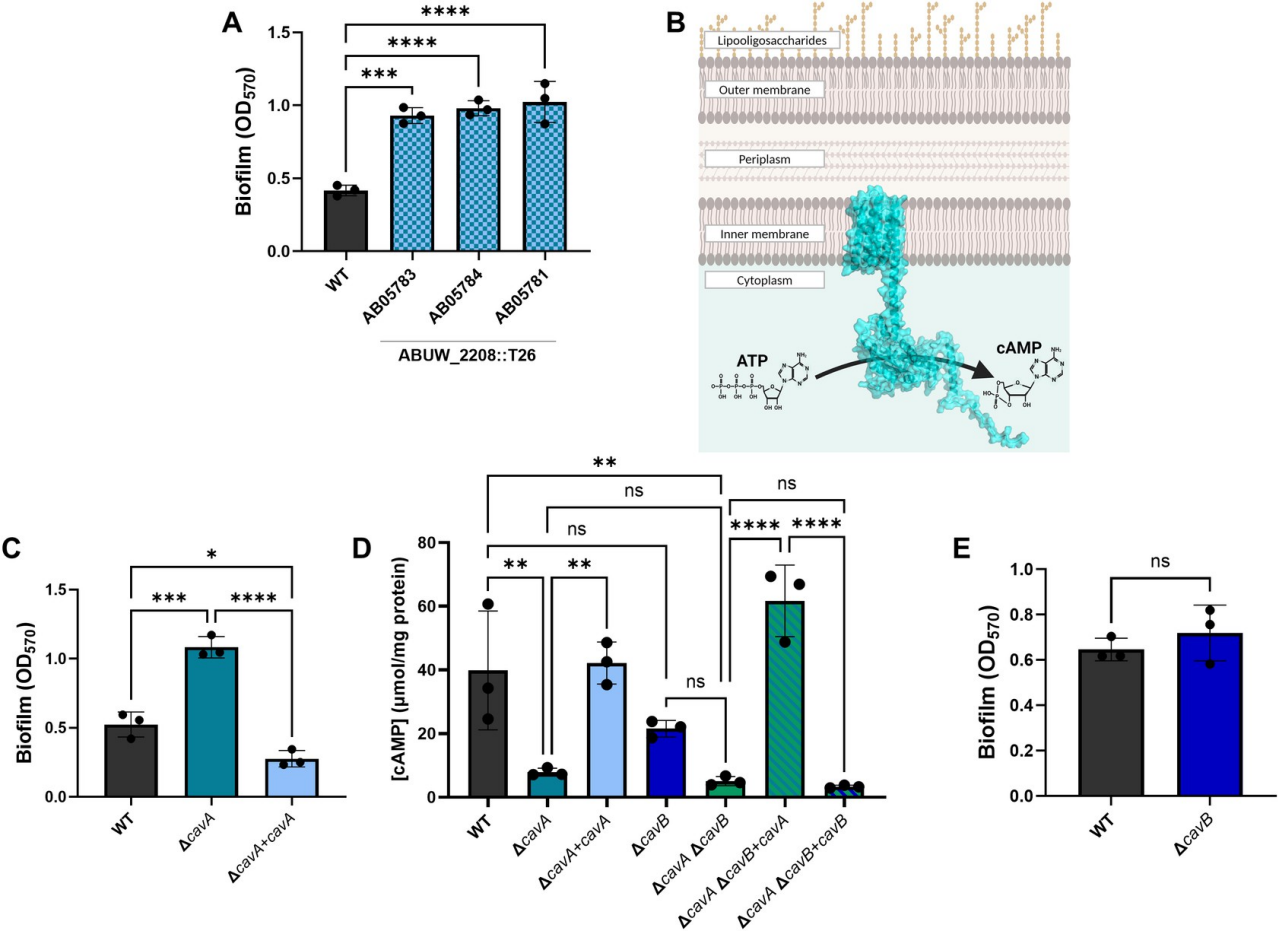
CavA (*ABUW_2208*) is a regulator of biofilm formation and the main functional adenylate cyclase in *A. baumannii*. **A—**Biofilm formation of wild-type (WT) AB5075 compared to three *ABUW_2208*::T26 transposon mutants labelled with their corresponding number from the Manoil transposon mutant library. **B**—Proposed cell localisation and function of CavA adenylate cyclase based on domains present in the protein. The transmembrane domains anchor the protein to the inner membrane the cells. The catalytic domain forms a dimer and converts ATP molecules to cyclic AMP (cAMP). Figure was created using PyMOL and BioRender.com. **C**–Biofilm levels of Δ*cavA* clean deletion mutant and Δ*cavA*+*cavA* complemented strain. **D**–cAMP concentrations in Δ*cavA* mutant and complemented Δ*cavA*+*cavA* strain, as well as Δ*cavB* and Δ*cavA*Δ*cavB* double mutant which was complemented with either *cavA* (Δ*cavA*Δ*cavB*+*cavA*) or *cavB* (Δ*cavA*Δ*cavB*+*cavB*). Cultures grown in LB broth were harvested and lysed via sonication. Bradford assay was used to quantify the total protein concentration and cAMP concentrations in each strain were determined using Cyclic Nucleotide XP Enzymatic Immunoassay kit. Data shown is the mean cAMP concentration per milligram protein from three independent repeats. **E—**Biofilm formation of Δ*cavB* deleted mutant compared to WT. Biofilm biomass was assessed after 24 h incubation at 37°C shaking and is presented as the optical density measured at 570 nm (OD_570_). All data represents the averages of three biological replicates ± standard deviations (SD). Bacterial growth was assessed at OD_600_ prior to staining the biofilms (S1 Fig). ns p>0.05, *p<0.05, ** p<0.01, *** p<0.001, **** p<0.0001—One-Way ANOVA with Dunnett (**A**) or Tukey (**C, D**) post-hoc tests and Unpaired t-test (**D**). Strains with empty miniTn7 were used as controls (S1 Fig).

*A*. *baumannii* is able to form robust biofilms that help it to colonise surfaces and tolerate antibiotic exposure. However, the mechanisms behind the regulation of biofilm formation in this pathogen are not fully understood. To identify biofilm regulators, we screened the Manoil *A*. *baumannii* AB5075 transposon mutant library to identify mutants with an altered biofilm phenotype [24]. After screening more than 10,600 mutants, amongst the hits were three different transposon mutants, bearing independent insertions at different positions within the same gene, *ABUW_2208*, a predicted adenylate/guanylate cyclase. To further validate the phenotype, all three *ABUW_2208*::T26 transposon mutants were tested for biofilm formation using a standard microtiter dish assay (Fig 1A). The transposon mutant strains AB05781, AB05784 and AB05783 had significantly higher biofilm biomass than the wild-type (WT) AB5075 (Fig 1A) validating the findings of the screen. Amplification of *ABUW_2208* gene by PCR confirmed the T26 transposon insertion in the gene in each of the three strains (S1A Fig). This indicated that a dysfunctional *ABUW_2208* enhanced *A*. *baumannii* biofilm formation, highlighting it as the most reproducible candidate gene and a potent negative regulator of this phenotype.

### *ABUW_2208* is the primary functional adenylate cyclase in AB5075

To understand the functional role of *ABUW_2208*, we investigated its predicted structure and predicted cellular localisation. We found that *ABUW_2208* (489 residues) is the only annotated adenylate/guanylate cyclase (AKA31936.1) in *A*. *baumannii* AB5075-UW genome (CP008706.1) and is annotated as CyaA in UniProt (A0A059ZQ67). Protein sequence analysis showed that, towards its N-terminus, *ABUW_2208* contains several regions predicted to be embedded in the membrane (S1B Fig). Towards its C-terminus, there is an Adenylate and Guanylate cyclase catalytic domain (PF00211) which is part of class III nucleotidyl cyclases and is predicted to dimerise. It contains a metal-binding site, as well as an active site where the conversion of ATP to cAMP occurs (Fig 1B). On the other hand, CyaA is a cytoplasmic class I AC in *P*. *aeruginosa* [25]. Therefore, according to its domain architecture, it would be inappropriate to refer to *ABUW_2208* as CyaA. Thus, due to its putative **c**yclase function in ***A***. *baumannii* and role in **v**irulence, we renamed *ABUW_2208* to CavA.

In order to eliminate the possibility of polar effects or off-target transposon insertions in the validated *cavA*::T26 mutants that may affect their biofilm phenotype, a clean deletion mutant and complemented strain were constructed using a previously developed genome editing strategy for MDR clinical isolates of *A*. *baumannii* [26]. As observed with the transposon mutants (Fig 1A), Δ*cavA* produced significantly more biofilm than the wild-type AB5075 (Fig 1C). The effect on the phenotype was reversed upon chromosomal complementation of the Δ*cavA* mutant, where Δ*cavA*+*cavA* biofilm levels were significantly lower than those of the deleted mutant and the WT (Fig 1C). The miniTn7 empty vector used for the complementation of the mutant was inserted in the WT AB5075 and Δ*cavA* and had no effect on the bacterial growth or biofilm biomass (S1C and S1D Fig). These results undoubtedly show that CavA plays a key role in the regulation of biofilm formation in *A*. *baumannii*.

We next sought to confirm if CavA was indeed a functional adenylate cyclase by measuring the cAMP levels in the deletion mutant compared to the WT and complemented strains. The Δ*cavA* mutant had the expected significantly lower cAMP levels than the WT (Fig 1D). This was reversed and cAMP concentrations were brought back to WT levels upon complementation in Δ*cavA*+*cavA* strain (Fig 1D), confirming the adenylate cyclase function of CavA enzyme. In addition, our data showed that expressing *cavA* in the WT background (WT+*cavA*) did not significantly increase cAMP concentrations than the WT (S1E Fig). However, as the deletion of *cavA* would drop the cAMP concentration compared to the WT, this suggests that under the conditions tested *cavA* is constitutively functional, leading to high basal cAMP levels in AB5075. Overall, our data demonstrates for the first time that there is a direct link between cAMP levels and surface associated biofilm formation in MDR *A*. *baumannii*.

Apart from *cavA*, which was annotated as an adenylate/guanylate cyclase, there is another adenylate cyclase annotated in the AB5075 genome within the *ABUW_1085* locus (AKA30837.1). In agreement with the *cavA* renaming, we renamed it to *cavB*. Despite their similar size and functional annotation, no significant similarity was found when CavA and CavB protein sequences were aligned. Unlike CavA, CavB is not predicted to have any transmembrane domains and has a CYTH domain (PF01928) with an active site and metal binding sites near its N-terminus, as well as a CHAD domain (PF05235) near the C-terminus (S1F Fig). Nevertheless, according to their annotation, it is predicted that CavB function is similar to that of CavA in synthesising cAMP. To assess this, we created a *cavB* clean deletion mutant as well as a double deletion mutant of *cavA* and *cavB* (Δ*cavA*Δ*cavB*) utilising the same strategy as before [26] and tested the cAMP concentrations and biofilm levels. The results demonstrated that deletion of *cavB* did not significantly alter cAMP concentrations compared to the WT (Fig 1D). In addition, the absence of *cavB* in the Δ*cavA* background (Δ*cavA*Δ*cavB*) did not decrease the cAMP concentrations further than the single *cavA* mutant (Δ*cavA*). Complementation of the double Δ*cavA*Δ*cavB* mutant with *cavA*, but not with *cavB*, restored cAMP concentrations to levels comparable to the WT (Fig 1D). This clearly indicates that, under the conditions tested, CavA is the only functional adenylate cyclase in *A*. *baumannii*. Thus, in agreement with our results presented above, linking biofilm formation and cAMP levels, the deletion of *cavB* did not alter bacterial growth (S1G Fig) or biofilm formation in comparison to the WT (Fig 1E). This confirms the major role of CavA in cAMP production and biofilm formation regulation of *A*. *baumannii*

In order to assess the prevalence of CavA and CavB, we analysed the frequency of all possible CavA and CavB variants across 9,696 *A*. *baumannii* genomes. Our findings revealed that both proteins are highly conserved, but between the two, CavA is less likely to undergo mutations, as fewer variants were identified of this protein compared to CavB (S2A Fig). This was observed regardless of the isolation site of the strains analysed, reinforcing the hypothesis of the major regulatory role of CavA (S2B Fig). In addition, CavB appears to have a different variant in environmental isolates–only 25% have the most frequent variant in humans and hospitals (S2B Fig).

### cAMP mediates transcription of various virulence-related genes in *A*. *baumannii*

**Fig 2:**
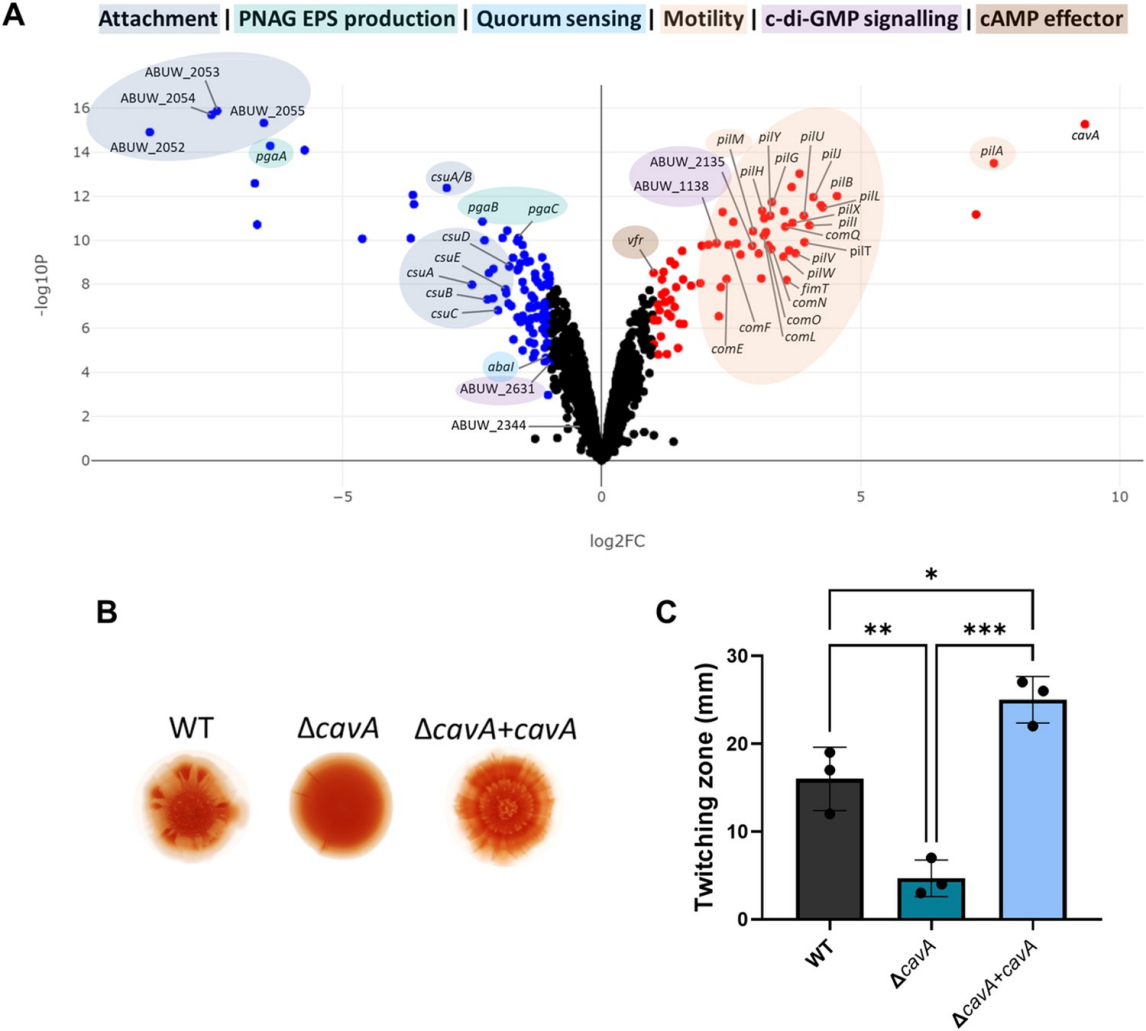
The effect of CavA and cAMP on *A. baumannii* transcriptome, EPS production and motility. **A**—Volcano plot showing dRNA-seq data of global transcription of Δ*cavA*+*cavA* complemented strain (having high cAMP levels) compared to the control Δ*cavA* EV deleted mutant with empty vector (having low cAMP levels). The results indicated 234 differentially expressed genes in total, of which 143 genes were up-regulated (red dots) and 91 were down-regulated (blue dots). Highlighted are up-regulated genes linked to twitching motility (pil and com gene clusters), cAMP signalling (*vfr*) and c-di-GMP synthesis (*ABUW_2135*) and degradation (*ABUW_1138*) and down-regulated genes linked to biofilm formation and attachment (*ABUW_2052–55* and *csu* operons), exopolysaccharides production (*pga* gene cluster), quorum sensing (*abaI*), c-di-GMP degradation (*ABUW_2631*). The *crp* homologue *ABUW_2344* was amongst the not significantly differentially expressed genes (black dots). **B** & **C**—The impact of different cAMP levels in Δ*cavA* mutant and complemented Δ*cavA*+*cavA* strain compared to wild-type (WT) AB5075 on exopolysaccharide (EPS) production (**B**) and twitching motility (**C**). For the EPS assay colonies of each strain were grown in triplicates on Congo red (40 μg/ml) agar plates at 37°C for 5 days and the images shown are representatives of three independent repeats (**B**). The twitching zone diameter was measured in millimetres 48 h post-inoculation of soft agar plates. Data represents the mean ± SD of three biological repeats. *p<0.05, **p<0.01, ***p<0.001, ****p<0.0001—One-Way ANOVA with Tukey post-hoc test. Strains with empty miniTn7 were used as controls in both assessments (S3A and S4A Figs).

cAMP is a second messenger known to act as a central signalling molecule in other species, regulating multiple processes simultaneously. To unravel what other phenotypes, apart from biofilm formation, could be regulated by cAMP, we performed a differential RNA sequencing (dRNA-seq) analysis of the Δ*cavA* mutant compared to the complemented mutant strain (i.e. with the gene reintroduced in a neutral chromosomal site). Cells were grown to mid-log phase before RNA extraction and subsequent sequencing and comparison. Our results show that 234 genes were differentially expressed by |Log2(Fold Change)| ≥ 1 in high cAMP conditions (Δ*cavA*+*cavA*) compared to low cAMP conditions (Δ*cavA* empty miniTn7 mutant) (S3 Table). Of these genes 143 were up-regulated and 91 were down-regulated (Fig 2A). To elucidate the main functional gene classes among the differentially expressed genes by cAMP, we performed a Gene Set Enrichment Analysis (GSEA) using FUNAGE-Pro [27]. The GSEA results revealed that cell adhesion and type IV pilus-mediated motility were affected (S4 Table). Our dRNA-seq data showed that genes linked to biofilm formation such as *csuA/BABCDE* operon, fimbriae coding genes (*ABUW_2052–2055*) and EPS production related genes (*pgaABC*) were down-regulated by cAMP. Simultaneously the expression of type IV pili genes (*pil* and *com* gene clusters), involved in twitching motility and natural transformation, was increased by this second messenger (Fig 2A). Altogether, our dRNA-seq dataset shows that cAMP acts as a global signalling molecule orchestrating antagonistic cell behaviours and virulence factors in *A*. *baumannii*, such as biofilm formation and motility, and multiple sub-processes that are involved in them.

### cAMP inversely regulates biofilm and motility in *A*. *baumannii*

EPS is a major component of bacterial biofilm extracellular matrix [28]. As indicated by the dRNA-seq, expression of the *pgaABCD* operon, encoding the synthesis machinery for the major exopolysaccharide poly-β-1,6-N-acetylglucosamine (PNAG) in *A*. *baumannii* [29], is significantly decreased in high cAMP conditions (Fig 2A). To phenotypically validate this link between cAMP and EPS production, the CavA-related strains were grown on Congo red agar plates for 5 days. As seen in this result (Fig 2B), and in accordance with the transcriptomics results, Δ*cavA* mutant colonies accumulated more Congo red stain than the WT colonies, which indicates a greater production of EPS. This phenotype was reversed upon complementation in Δ*cavA*+*cavA* strain (Fig 2B), as the empty vector used for complementation did not affect EPS production (S3A Fig). These results further support our data presented above indicating the negative impact of cAMP on *A*. *baumannii* biofilm formation (Fig 1C). To validate that the effect of CavA on EPS production was attributed to the differential regulation of the *pgaABCD* operon and PNAG production, a Δ*cavA*/*pgaA*::Tn double mutant was created. The results showed a substantial decrease in the accumulated Congo red stain in the double mutant colonies compared to the single Δ*cavA* mutant (S3B Fig). Hence, cAMP represses expression of EPS related genes, resulting in decreased EPS production by *A*. *baumannii*. Further to this, assessment of Δ*cavA*/*pgaA*::Tn and Δ*cavA*/*csuC*::Tn double mutants showed significant decrease in the elevated biofilm levels caused by the deletion of *cavA* (S3C and S3D Fig). However, disruption of *pgaA*, or *csuC*, in a *cavA* mutant, did not decrease the high biofilm biomass to WT levels. This suggests the cumulative effect of the increased expression levels of these genes underpins the increased biofilm formation seen in the Δ*cavA* mutant. Our data highlights that cAMP control of biofilm formation is multifactorial and involves transcriptional regulation of different genes including *csu* and EPS production related genes.

Biofilm formation and twitching motility are known to be inversely regulated antagonistic lifestyles [20]. In the experiments above, we have established that high cAMP negatively regulates biofilm formation (Figs 1C and 2B). This, together with our findings showing an up-regulation of the type IV pili related genes in high cAMP conditions (Fig 2A), strongly indicated that this second messenger could play a role in the regulation of twitching motility in *A*. *baumannii*. In order to validate this cAMP regulation, we assessed *A*. *baumannii* motility in the Δ*cavA* mutant compared to the wild-type strain using a twitching assay (Fig 2C). In accordance with the dRNA-seq results, we observed significant decrease in Δ*cavA* mutant twitching motility. Chromosomal complementation of the deleted *cavA* mutant restored motility (Fig 2C), as the empty miniTn7 vector did not affect the phenotype (S4A Fig). In addition to the impairment in twitching motility, we could also observe that the natural transformation capability of the Δ*cavA* mutant decreased below the detection limit of our natural transformation assay compared to the wild type (S4B Fig). This clearly demonstrates the key role of cAMP in promoting transformability and motility in *A*. *baumannii* via activation of type IV pili gene expression to the detriment of biofilm formation.

### Vfr regulates biofilm, motility and EPS in a cAMP-dependent manner in *A*. *baumannii*

In other bacterial species, such as *Pseudomonas aeruginosa* and *Escherichia coli*, several cAMP effector proteins have been identified, including Vfr and CRP [30]. Upon binding to cAMP, Vfr undergoes a conformational change shifting this transcriptional factor from an inactive to active state [31]. In the case of *A*. *baumannii* AB5075, a *vfr* orthologue is annotated in its genome (*ABUW_2741* locus, AKA32458.1), but the link between Vfr and cAMP signalling has not been previously established in this pathogen. Moreover, there is also a predicted *crp* orthologue annotated in AB5075 (*ABUW_2344* locus, AKA32071.1) with a predicted cAMP-binding domain. Within our transcriptomic dataset, we identified that *vfr* expression was significantly increased (Log2(FC) = 1) in high cAMP conditions, whereas the predicted *crp* gene was not differentially expressed (Fig 2A). To obtain evidence that the AB5075 Vfr orthologue may bind cAMP, we compared its protein sequence to that of the *P*. *aeruginosa* PAO1 Vfr protein, for which the residues interacting with the cAMP molecule have been elucidated. As a result, we found that these residues are conserved, along with the DNA binding domain, thus hinting the link between Vfr and cAMP signalling in *A*. *baumannii* (S5A Fig). Moreover, alignment of Vfr^AB5075^ and Vfr^PAO1^ protein structure models resulted in a root mean square deviation (RMSD) of 1.94 indicating the structural similarity of the two proteins (S5B–S5D Fig). Furthermore, we analysed the conservation of Vfr across the *A*. *baumannii* pangenome, obtaining as a result that Vfr was present in 81% of the strains (S2A Fig). In contrast, a small proportion (19%) of the strains did not have Vfr, suggesting a possible role of additional alternate cAMP effectors, which requires further investigation. The majority of the strains had only two Vfr sequence variants, indicating a strong negative selection. Additionally, CavA and Vfr present co-conservation (maintaining the same pair of variants in the same strain) in the majority of the analysed strains (S2A Fig), suggesting a link between them in the physiology of *A*. *baumannii*.

**Fig 3:**
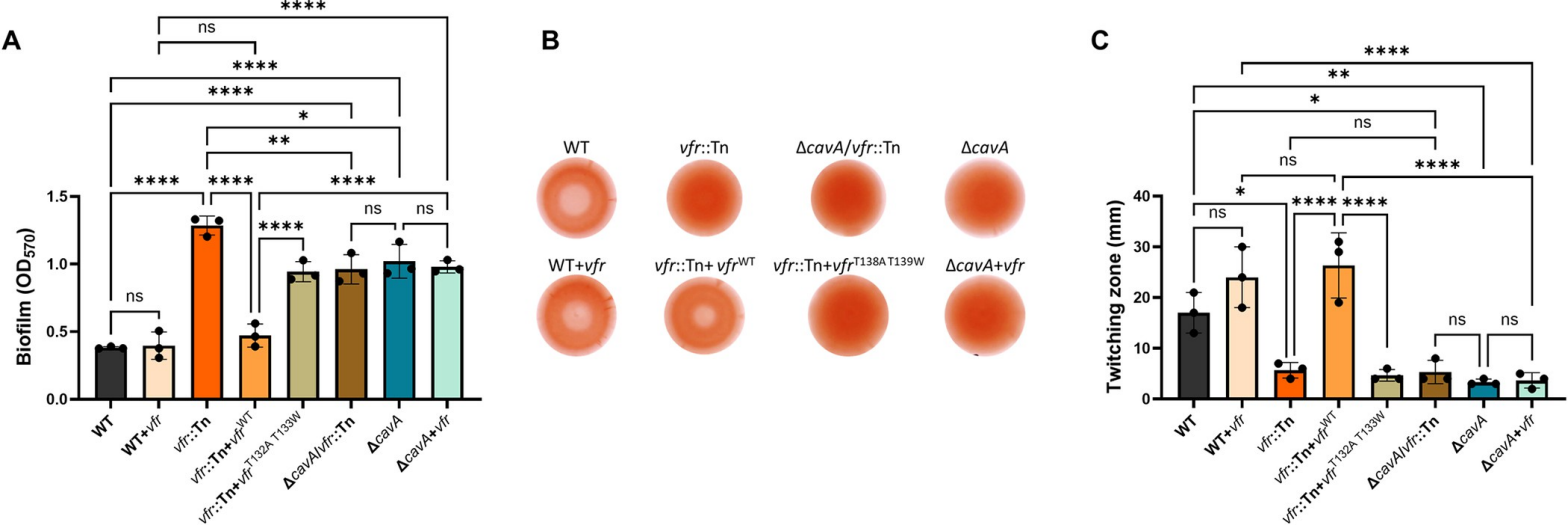
Vfr negatively regulates biofilm formation, EPS production and motility of *A. baumannii* in a cAMP-dependent manner. Biofilm formation (**A**), EPS production (**B**) and twitching motility (**C**) of *vfr*::Tn transposon mutant AB07171, its complemented strain *vfr*::Tn+*vfr*^WT^, strains overexpressing *vfr* in the WT (WT+*vfr*) and Δ*cavA* backgrounds (Δ*cavA*+*vfr*) compared to AB5075 WT. In addition, *vfr*::Tn mutant was complemented with *vfr* variant harbouring two modified residues (T138A and T139W) in the cAMP binding site (*vfr*::Tn+*vfr*^T138A,T139W^). **A**—Biofilm formation microtiter assay was used as described above and biofilms were grown in LB broth for 24 h at 37°C shaking. Growth of the strains was assessed and strains harbouring empty miniTn7 were used as control. **B**—Images of colonies on Congo red agar for EPS production assessment were taken 5 days post-inoculation. **C**—Motility was assessed using soft agar at 48 h as described in the materials and methods section. Data including all controls is presented in S6 Fig. All experiments were repeated three independent times. Bar graphs present averages ± SD. *p<0.05, **p<0.01, ***p<0.001, ****p<0.0001—One-Way ANOVA with Dunnet (**A**) and Tukey (**B** & **D**) post-hoc tests.

To establish the role of Vfr as a cAMP effector, we first validated the transposon insertion in *vfr*::Tn (AB07172) transposon mutant via Sanger sequencing. We then complemented this mutant and verified that neither of the *vfr*-related derivatives had impaired growth and that the miniTn7 empty vector did not affect the tested phenotypes (S6 Fig). The elevated biofilm phenotype of the *vfr*::Tn mutant was reversed upon chromosomal complementation with *vfr*^WT^ (Fig 3A). To verify the cAMP control of Vfr, we generated a Vfr variant with point mutations in two key cAMP-binding residues (T138A and T139W) in the C-helix of the protein (S5E Fig), which is known to act as the activation mechanism of Vfr in *P*. *aeruginosa* [31]. In contrast to *vfr*^WT^, *vfr*^T138A,T139W^ variant was impaired for complementation of the *vfr*::Tn mutant biofilm phenotype. Ectopic expression of *vfr* in the WT (WT+*vfr*) did not change biofilm levels compared to the WT and expression of *vfr* in Δ*cavA* mutant (Δ*cavA*+*vfr*) did not alter the high biofilm levels produced by Δ*cavA* (Fig 3A). These findings confirm that Vfr acts downstream of CavA on biofilm formation and that its function is cAMP mediated which is in accordance with what is seen in other bacteria [31].

The fact that Vfr channels the CavA-mediated cAMP effect on biofilm formation made us hypothesize whether Vfr may be involved in other CavA-controlled behaviours, including EPS production and twitching motility. Further exploration of this regulation showed that *vfr*::Tn exhibited an increased EPS production similarly to the Δ*cavA* mutant (Fig 3B). As observed with biofilm formation, EPS production decreased when the *vfr*::Tn mutant was complemented with *vfr*^WT^ (*vfr*::Tn+*vfr*^WT^), but not with *vfr*^T138A,T139W^ variant (*vfr*::Tn+*vfr*^T138A,T139W^). Also, the increased EPS production by Δ*cavA* was unaffected when *vfr* expression was enhanced in this background (Δ*cavA*+*vfr*). Furthermore, we assessed twitching motility in the *vfr*::Tn mutant compared to the WT and the Δ*cavA* mutant and determined that *vfr*::Tn has a similar twitching profile to that of the Δ*cavA* mutant, with a significant decrease in motility compared to the WT (Fig 3C). Complementation of the *vfr*::Tn strain with *vfr*^WT^, but not with *vfr*^T138A,T139W^, restored motility to WT levels. Similarly to our observations on the biofilm formation (Fig 3A), increased expression of *vfr* in the WT (WT+*vfr*) or Δ*cavA* mutant background (Δ*cavA*+*vfr*) phenocopied WT and Δ*cavA* motility respectively (Fig 3C). Additionally, disruption of *vfr* in the double Δ*cavA*/*vfr*::Tn mutant did not further alter the Δ*cavA* biofilm formation, EPS production and motility phenotypes (Fig 3). This was also confirmed by assessing the expression of *pilA* gene and *pgaABCD* operon using *PpilA*::*gfpmut3* and *Ppga*::*gfpmut3* transcriptional fusions respectively. Our data showed that the expression of *pilA* and *pgaABCD* was significantly decreased in *vfr*::*Tn* mutant as well as Δ*cavA* compared to the WT (S7 Fig).

Altogether, these findings confirm that Vfr function is dependent on the presence of the major adenylate cyclase in *A*. *baumannii*, CavA, and highlight the cAMP specificity in Vfr functionality. This clearly demonstrates that, similarly to *P*. *aeruginosa* [31], Vfr acts as a cAMP effector in *A*. *baumannii* as well.

### cAMP negatively regulates QS via *abaI* expression

**Fig 4:**
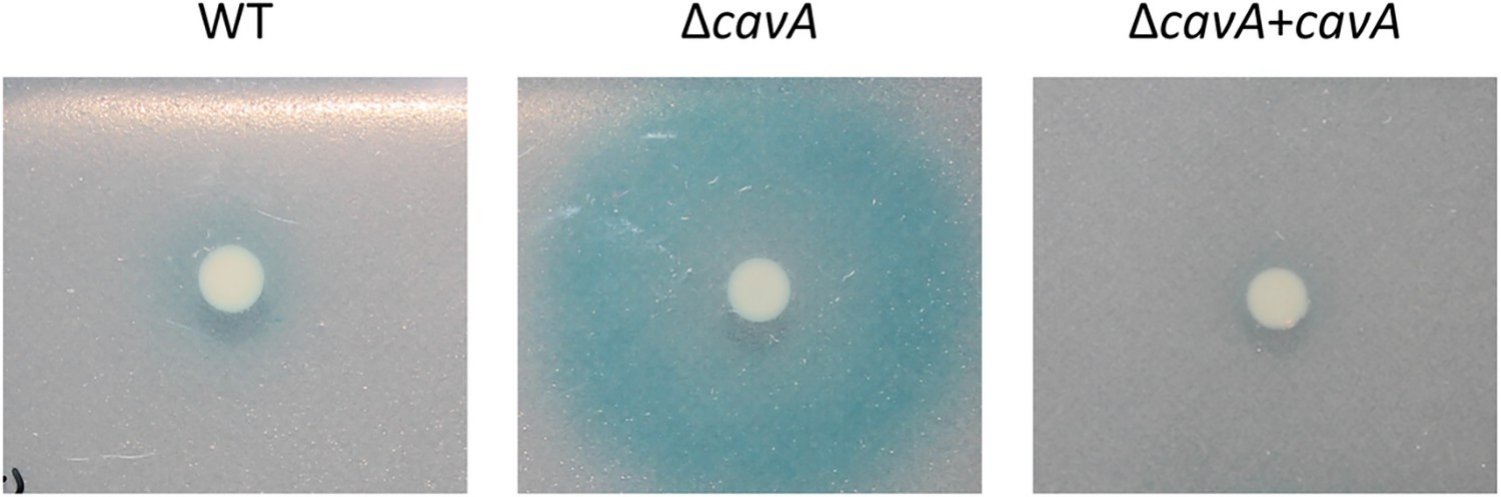
Qualitative assessment of AHL secretion by AV-T variants of WT *A*. *baumannii* AB5075, deleted Δ*cavA* mutant and Δ*cavA*+*cavA* complemented strain. Cultures of each strain were spot plated on a soft agar mixed with *A. tumefaciens traG-lacZ* biosensor strain and supplemented with X-Gal and IPTG. The blue halo indicates AHL production by *A*. *baumannii* which triggers the cleavage of X-gal by the *A*. *tumefaciens* biosensor strain. Empty miniTn7 controls are presented in S8 Fig.

Another interesting observation from our dRNA-seq dataset was that the sole QS autoinducer synthase gene encoded in AB5075, *abaI*, had decreased expression in high cAMP conditions (Fig 2A). Similarly to observations in other bacterial species where a link between second messenger and quorum sensing signalling systems have been reposted [32,33], our dRNA-seq data suggests that there may be an interplay between intracellular second messenger signalling and intercellular communication in *A*. *baumannii* too. To explore this further, we sought to verify the AHL production, and thus the QS regulation, by CavA at the phenotypic level using an *Agrobacterium tumefaciens* AHL biosensor strain (Figs 4 and S8). We could indeed observe that the Δ*cavA* mutant produced greater amounts of AHL compared to the WT strain, indicating that cAMP is able to reduce QS via transcriptional regulation of *abaI* (Fig 4). Coherently, the complementation of the *cavA* mutation abolished the AHL production restoring the WT phenotype. Together, this conclusively indicates that the CavA-mediated cAMP second messenger signalling is able to modulate a primary intercellular communication system as QS in *A*. *baumannii*.

### cAMP regulates c-di-GMP Levels in *A*. *baumannii*

Second messengers, as signalling molecules, are known to overlay their regulatory targets to modulate the global phenotypic output of the cell. One well-established model in the bacterial cell signalling field is that high cAMP levels correlate with high motility and low biofilm formation (planktonic lifestyle), whereas high c-di-GMP levels are associated with low motility and high biofilm formation (sessile lifestyle) [34].

Our dRNA-seq dataset highlighted that the expression of several genes, that encode both predicted diguanylate cyclases and phosphodiesterases, was increased in high cAMP conditions. This includes the DGC *ABUW_2135* and the PDE *ABUW_1138* (Fig 2A). We could also observe a decreased expression of the predicted PDE *ABUW_2631*, however, this is annotated as a pseudogene in the reference AB5075 genome [24], hinting it would probably yield a non-functional protein. Nevertheless, although DGCs and PDEs have opposite effects on c-di-GMP concentrations, our transcriptomics dataset suggested that cAMP may have an effect on the c-di-GMP levels.

To test this, we aimed to quantify the c-di-GMP levels in our mutant and complemented strains, as well as in the WT. To perform these assays, we adapted the previously reported and validated CensYBL c-di-GMP fluorescent biosensor [35] for its use in *Acinetobacter*. This biosensor is based on the c-di-GMP binding protein BldD, which is able to dimerise via interaction with this second messenger molecule. In the CensYBL biosensor, two copies of the *bldD* genes are fused to the 5’ and 3’ halves of the fluorescent protein coding gene *yfp*. After their expression, the BldD fragments of the N-YFP::BldD and C-YFP::BldD fusion proteins will dimerise in the presence of c-di-GMP. This would reconstitute the YFP function, thus producing a level of fluorescence that is proportional to the c-di-GMP levels in the cell. We adapted this biosensor to *Acinetobacter* by assembling the construct into an *Acinetobacter*-compatible pWH1266 derivative plasmid (pWH1266-Apr). Furthermore, we cloned the biosensor coding genes downstream of an anhydrotetracycline-inducible promoter. With these modifications, we could not only use the biosensor in any *Acinetobacter* species, but more specifically, we could select it in any mutant from the Manoil transposon mutant library and induce the biosensor independently of the set of expression systems we routinely use for gene complementation (IPTG-inducible). To validate the functionality of the modified CensYBL biosensor (CensYBL-Ab) in *Acinetobacter*, we introduced the heterologous DGC coding gene pleD* and the PDE *rocR* under an IPTG-inducible promoter in AB5075 via miniTn7 insertion. As a result, we could validate our modified c-di-GMP biosensor by measuring a signal increase or decrease after the induction of pleD* or *rocR*, respectively, compared to an empty vector control (S9A Fig).

**Fig 5:**
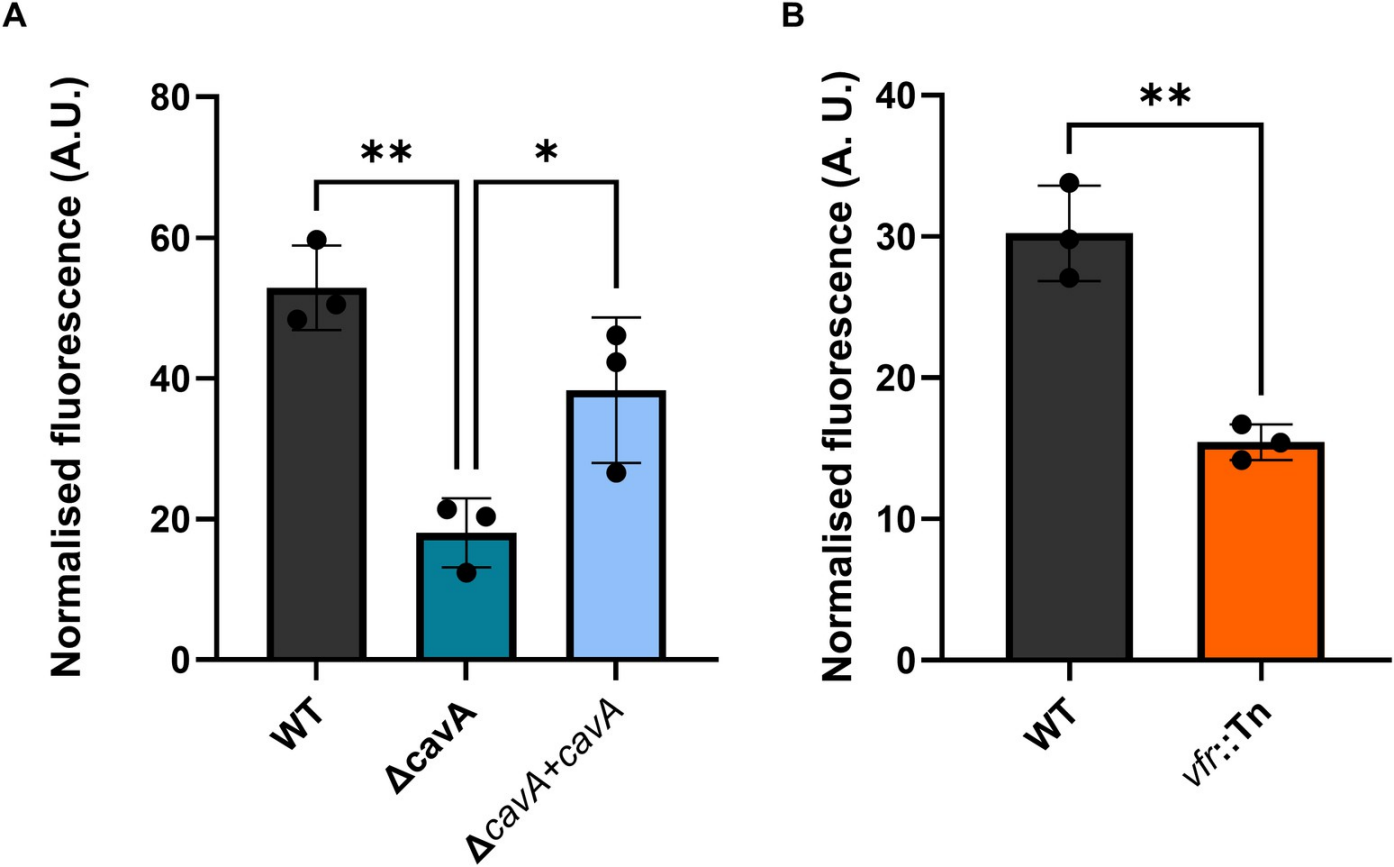
CavA and Vfr are regulators of global intracellular c-di-GMP levels in *A. baumannii*. Cyclic di-GMP levels measured using CensYBL-Ab biosensor in Δ*cavA* mutant with low cAMP and Δ*cavA*+*cavA* complemented strain with high cAMP levels (**A**) as well as *vfr*::Tn mutant (**B**) compared to WT AB5075. Deletion of *cavA* reduced c-di-GMP levels by 66% (**A**) while disruption of *vfr* decreased them by 49% (**B**). Cultures were diluted in LB broth supplemented with apramycin (100 μg/ml) and biosensor expression was induced with anhydrotetracycline (50 ng/ml). Harvested cells were resuspended in sterile PBS and YFP and mCherry fluorescence signals were measured. The average normalised fluorescence (YFP/mCherry) ± SD from three independent biological repeats is presented. Data was analysed using One-Way ANOVA with Tukey post-hoc test (**A**) and Unpaired t-test (**B**) (* p<0.05, ** p<0.01). Strains harbouring empty miniTn7 were used as controls (S9B Fig).

In order to test if cAMP levels affect the global c-di-GMP levels in AB5075, we introduced our CensYBL-Ab biosensor in the Δ*cavA* mutant, as well as in the transposon mutant in the cAMP effector *vfr*. Strikingly, absence of CavA (Fig 5A) or Vfr (Fig 5B) significantly decreased c-di-GMP levels compared to the WT, with c-di-GMP reduction levels in the Δ*cavA* and *vfr*::Tn mutants of 66% and 49% respectively. The c-di-GMP levels were partially restored in the Δ*cavA*+*cavA* strain upon *cavA* complementation, as the empty miniTn7 vector used for the complementation had no effect (S9B Fig). Our data demonstrates that the CavA-Vfr regulatory cascade modulates the c-di-GMP levels in *A*. *baumannii*. From a broader perspective, our results challenge a previously established paradigm, presenting cAMP and c-di-GMP as antagonistic and inversely regulated signalling molecules. Instead, our findings demonstrate that the c-di-GMP levels are not only positively regulated by cAMP, but also suggest that they are possibly hierarchically under the control of the Vfr-mediated cAMP regulation.

### cAMP regulates *A*. *baumannii* resistance to fosfomycin

**Fig 6:**
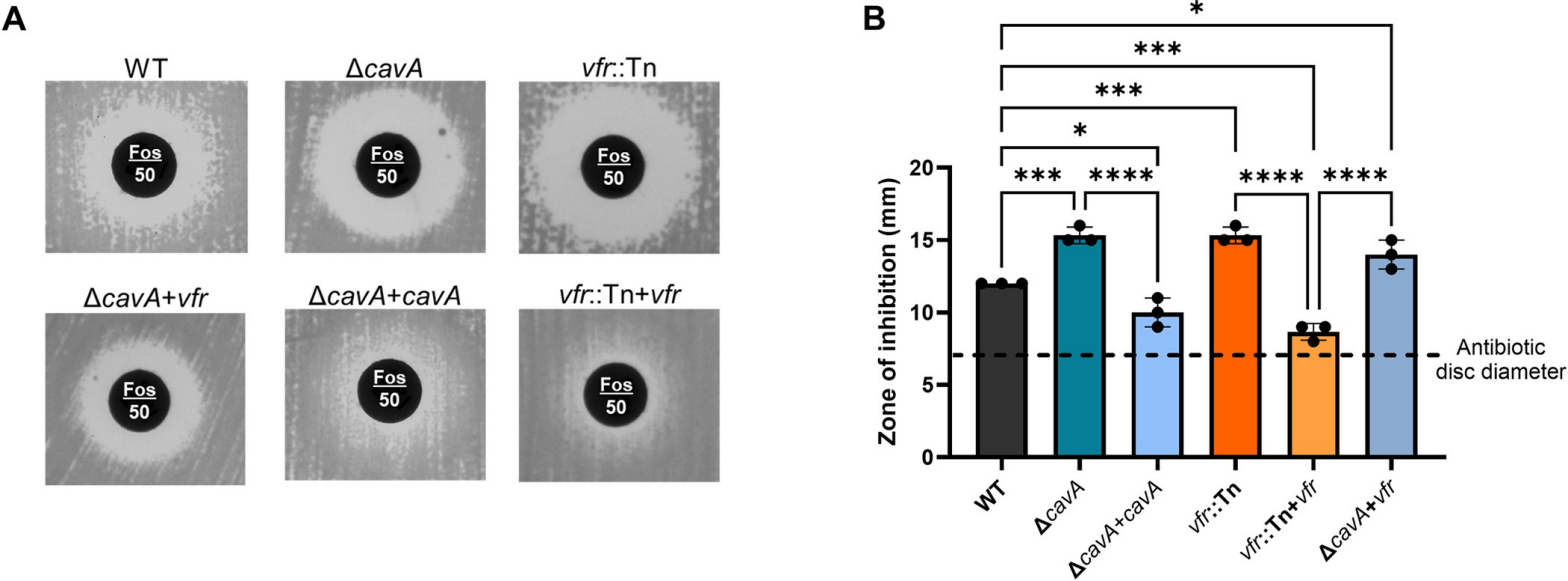
*A. baumannii* resistance to fosfomycin is regulated by CavA and Vfr. Disc diffusion assay for testing fosfomycin resistance of WT AB5075, Δ*cavA* deleted mutant, *vfr*::Tn transposon mutant and their complemented derivative strains (Δ*cavA*+*vfr* and *vfr*::Tn+*vfr* respectively), as well as Δ*cavA* mutant overexpressing vfr (Δ*cavA*+*vfr*). CAMH agar inoculated with each corresponding strain and having a fosfomycin (50 μg) disc were assessed after 24 h at 37°C. Representative images (**A**) and the measured zone of inhibition in millimetres (**B**) are shown. Data represents the averages ± SD of biological triplicates. ns p>0.05, *p<0.05, ***p<0.001, ****p<0.0001 –One-Way ANOVA with Dunnet post-hoc test (**B**). All controls used are presented in S10B Fig.

Given the role of antibiotic resistance in the ascendancy of *A*. *baumannii* to the top of the WHO priority pathogen list [36], we sought to assess if cAMP levels could influence antibiotic resistance in the MDR isolate AB5075. Initially we tested CavA role in *A*. *baumannii* resistance to different classes of antibiotics including quinolones (ciprofloxacin), polymyxins (colistin), amphenicols (chloramphenicol) and aminoglycosides (tobramycin) but we saw no significant difference between Δ*cavA* and WT (S10A Fig). It has been previously suggested that cAMP regulation could be involved in the antibiotic resistance phenotype of MDR *A*. *baumannii* clinical isolates. For example, mutations in *cavA* orthologous genes carried in *A*. *baumannii* clinical isolates genomes hinted a link to fosfomycin resistance [37]. However, a phenotypic link between cAMP and fosfomycin resistance has never been established in this pathogen. Hence, we sought to investigate this further using a disc diffusion assay. The results showed that lack of CavA, i.e. low cAMP conditions, or Vfr (in Δ*cavA* and *vfr*::Tn mutants respectively) significantly increased the zone of inhibition compared to the WT (Fig 6) and thus, increased AB5075 susceptibility to fosfomycin. This phenotype was reversed upon complementation in the Δ*cavA*+*cavA* and *vfr*::Tn+*vfr* strains (Figs 6 and S10B). As observed with other phenotypes, increased expression of *vfr* in the absence of CavA did not change the increased fosfomycin resistance of Δ*cavA* mutant (Fig 6). Altogether, this demonstrates that the cAMP singalling system may drive resistance to antibiotics, such as fosfomycin, in MDR *A*. *baumannii*. Furthermore, this cAMP-mediated regulation is exerted via Vfr.

### cAMP mediates *A*. *baumannii* virulence in *Galleria mellonella*

**Fig 7:**
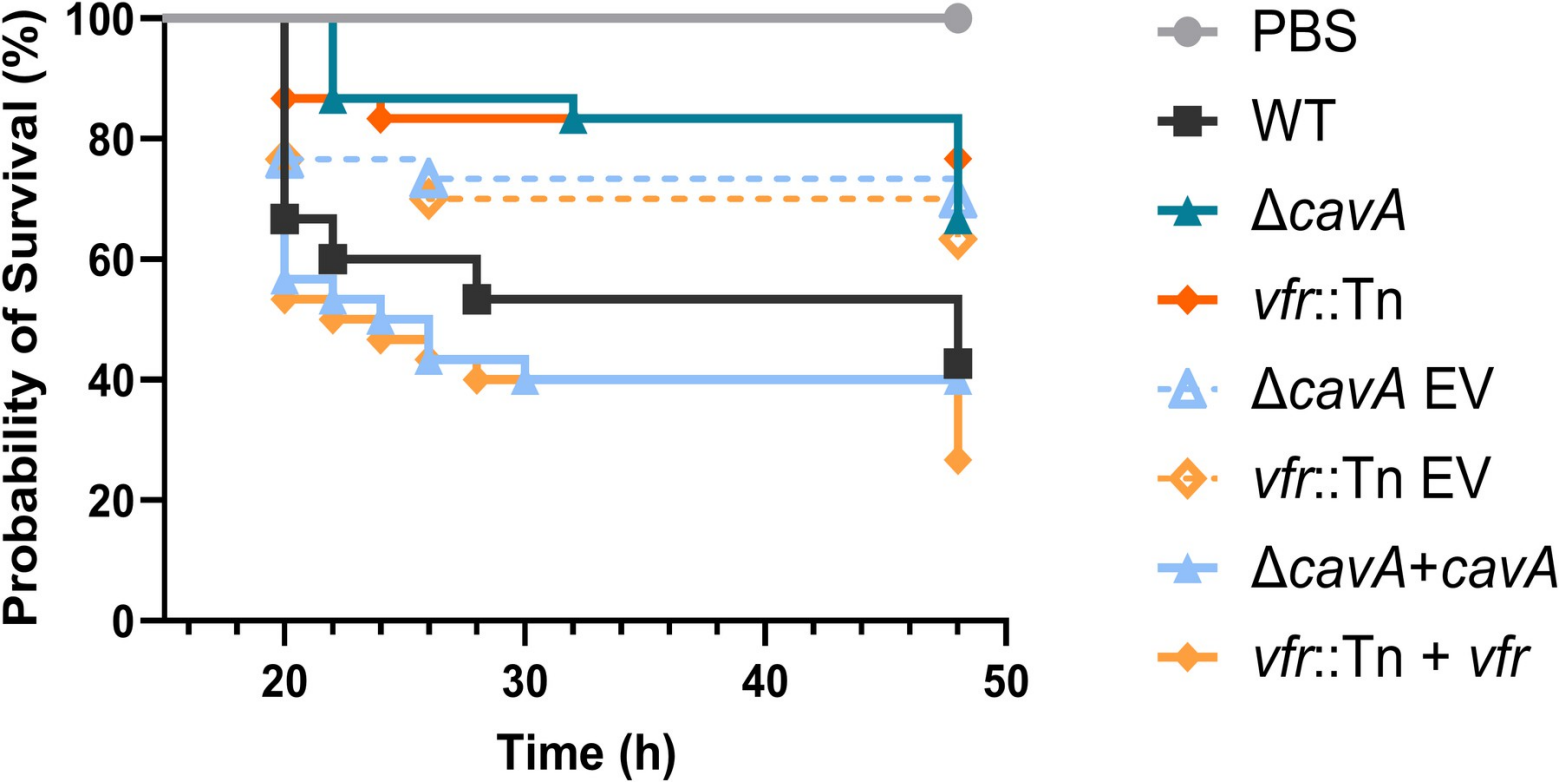
Cyclic AMP and Vfr increase the virulence of *A*. *baumannii*. Virulence assay was performed three independent times using 30 *G*. *mellonella* larvae in total per *A*. *baumannii* strain or control (PBS). 48 h post-infection, 100% of the larvae in the control group survived. *G*. *mellonella* infected with wild-type (WT) AB5075 had survival rates of 40% while 63% and 77% of the larvae survived infection with Δ*cavA* mutant and *vfr*::Tn mutant respectively. Survival rates of larvae infected with the complemented Δ*cavA*+*cavA* and *vfr*::Tn+*vfr* strains were decreased to 40% and 27% respectively. Strains bearing empty miniTn7 vector (EV) were used as controls. In the interest of visual clarity, the X axis (Time) was set to start at 15 h. Data analysis with Log rank (Mantel-Cox) test indicated p<0.05 for the comparisons of WT vs Δ*cavA* and Δ*cavA* EV vs Δ*cavA*+*cavA* and p<0.01 for the comparisons of WT vs *vfr*::Tn and *vfr*:Tn EV vs *vfr*::Tn+*vfr*.

As we have shown above, cAMP regulates virulence associated phenotypes such as biofilm formation and motility, and controls other signalling systems, all of which are related to *A*. *baumannii* virulence. In addition, genes related to twitching motility had increased expression in the presence of CavA (Fig 2A). As motility has been known to promote *A*. *baumannii* virulence [38], our transcriptomic data clearly suggested that reducing the cAMP levels (i.e. mutating *cavA*) would attenuate the virulence of *A*. *baumannii* AB5075. To confirm the role of cAMP in *A*. *baumannii* virulence regulation, we used the *G*. *mellonella in vivo* infection model. All larvae were infected with the WT or derivative mutants (a control group was injected with sterile PBS) and larvae survivability was assessed over two days. At 48 h post-infection 40% of larvae infected with WT AB5075 survived, whereas the survival rates were significantly increased in the groups infected with Δ*cavA* (with lower cAMP levels) and *vfr*::Tn mutants (63% and 77% respectively, Fig 7). Chromosomal complementation of *cavA* (Δ*cavA*+*cavA*) or *vfr* (*vfr*::Tn+*vfr*) significantly decreased survival rates (40% and 27% respectively) compared to the Δ*cavA* and *vfr*::Tn strains harbouring empty miniTn7 vector (EV) (70% and 63% respectively). These results demonstrate that high cAMP levels, and their Vfr-mediated regulation, are key for modulating the *in vivo* virulence of the priority pathogen *A*. *baumannii*.

## Discussion

The signalling cascade orchestrated by the universal second messenger cAMP plays a pivotal role in numerous aspects of bacterial physiology [30]. Amongst the functions regulated by cAMP in the well-studied pathogens *E*. *coli* and *P*. *aeruginosa*, there is virulence and related behaviours including the switch between motile and sessile lifestyles [30]. Despite *A*. *baumannii* being a top priority pathogen, information about second messenger signalling, and in particular cAMP signalling, in this pathogen is scarce. Here, we present the cAMP signalling cascade and a range of phenotypes controlled by it in the MDR *A*. *baumannii* AB5075. We demonstrate that cAMP promotes switching to motile lifestyle by decreasing EPS production and Csu pili-mediated attachment and increasing type IV pili at the transcriptional level, which leads to increased *A*. *baumannii* virulence. In addition, our data provides evidence that cAMP regulates fosfomycin resistance of this pathogen. Furthermore, the presented data suggests hierarchy of both intra- and intercellular signalling orchestrated by cAMP.

Our data highlighted CavA as the main AC responsible for the synthesis of the majority of cAMP, while CavB did not alter the global cAMP concentrations (Fig 1D). Furthermore, CavA but not CavB altered *A*. *baumannii* biofilm phenotype (Fig 1C and 1E), highlighting the role of CavA as the only functional AC under the conditions tested. Nevertheless, CavB is highly conserved across *A*. *baumannii* strains (S2A Fig), suggesting it may have a function in conditions different from the ones presented here.

In *P*. *aeruginosa* and *Vibrio cholerae*, cAMP is known to be a negative biofilm regulator [21,30]. In accordance with this, we show that cAMP has the same effect on *A*. *baumannii* biofilm formation in clinically relevant conditions (i.e. 37°C; Fig 1C). A previous report found that cAMP is needed for pellicle formation in a hypermotile variant of the lab adapted *A*. *baumannii* strain ATCC 17978 [18]. Pellicle formation is a type of biofilm that occurs at the air-liquid interface at 25°C [18] and thus, is relevant for *A*. *baumannii* environmental survival. This suggests that cAMP may mediate a temperature-dependent control of *A*. *baumannii* biofilm formation, which would aid its adaptation and survival within and outside the host.

Assessing the cAMP regulon using dRNA-seq uncovered the specific mechanisms through which it controls biofilm formation specifically via the decreased expression of genes encoding Csu pili and EPS production. These specific type I pili are crucial for *A*. *baumannii* biofilm formation, as well as its attachment to different abiotic and biotic surfaces [12,39]. Additionally, cAMP affects the biofilm extracellular matrix by reducing EPS production (Fig 2B) via transcriptional down-regulation of PNAG synthesis machinery coding genes (Figs 2A, S3B and S7). This cAMP-mediated biofilm formation is a result of a simultaneous global regulation of multiple biofilm related processes (Figs 2A and 3D). Moreover, sessility and motility are antagonistically regulated phenotypes [40]. In line with this, we observed that while decreasing sessility (Fig 1C), high cAMP promotes *A*. *baumannii* transition to motile lifestyle (Fig 2C) which is enhanced via increased expression of type IV pili related genes (Fig 2A). These behaviours contribute to define the virulence of *A*. *baumannii*. Indeed, we observed that cAMP promotes *A*. *baumannii* virulence in *G*. *mellonella* model (Fig 7). While other studies have shown that increased motility enhances *A*. *baumannii* virulence [38,41], this link has been predominantly related to surface-associated motility. On the other hand, our results provide an association between the cAMP-regulated twitching motility and *in vivo* virulence for the first time in *A*. *baumannii*. According to Ahmad *et al*. [12], Csu pili contribute to *A*. *baumannii* virulence by aiding attachment to epithelial cells and enhance lungs, spleen and liver colonisation in a mouse model. We observed that under high cAMP conditions, *A*. *baumannii* virulence was enhanced, despite the decreased expression of *csu* genes [12] suggesting that cAMP enhances motility of *A*. *baumannii* AB5075 and increases its virulence in a Csu-independent way. In addition to motility, the cAMP-mediated type IV pili regulation affected transformability (S4B Fig), highlighting the importance of the second messenger in the regulation of *A*. *baumannii* acquisition of foreign genetic material. These findings make cAMP regulation an appealing target for novel therapies against this pathogen.

One of the features that has made *A*. *baumannii* ascend to the top of the WHO priority pathogen list is its ability to acquire antibiotic resistance. A previous study showed that some *A*. *baumannii* clinical isolates with increased fosfomycin resistance had acquired mutations in CavA homologs amongst other proteins [37]. However, it was not established whether these mutations had an effect on the functionality of the AC or had a direct link to fosfomycin sensitivity as they were typically identified with several other mutations. The antibiotic sensitivity test we performed demonstrate that cAMP does indeed increase fosfomycin resistance of *A*. *baumannii* AB5075 (Fig 6). Our results are in accordance with other studies demonstrating similar control of resistance to this antibiotic by cAMP signalling in *E*. *coli* [42]. Fosfomycin is predominantly used for urinary tract infections (UTIs) [43,44] but has applications in treatment of infections beyond UTIs too [45]. It halts bacterial growth via inhibition of cell wall synthesis by targeting MurA [46]. *A*. *baumannii* fosfomycin resistance has been attributed to *abrp* gene and mutations in AbaF transporter [37,47]. Surprisingly, we did not see significantly altered expression of any of these genes, suggesting that cAMP may be regulating a novel fosfomycin resistance mechanism. Future work will focus on uncovering this resistance mechanism. However, given the capacity for temporal fluctuation of cAMP levels, it does suggest that manipulating cAMP levels may be a mechanism to confer adaptive fosfomycin resistance without the need for genetic mutation.

In order to elicit a response in the bacterial cell, second messenger molecules interact with effector proteins, such as Vfr [30]. However, there was no link established between cAMP and its effector protein in *A*. *baumannii*, despite the presence of the Vfr homolog. Based on the Vfr amino acid conservation and structural similarity between *P*. *aeruginosa* PAO1 and *A*. *baumannii* AB5075 (S5A–S5D Fig), we inferred that cAMP interaction with Vfr would occur similarly in both species. Also, the transcription of *vfr* is positively autoregulated in a cAMP-dependent manner in *P*. *aeruginosa* [48]. In accordance with this, we saw increased expression of *vfr* in elevated cAMP concentrations in AB5075 (Fig 2A). Furthermore, our phenotypic assessments showed that, in line with Δ*cavA* mutant (Figs 1C, 2B and 2C), Vfr had a negative effect on EPS production and biofilm formation, but enhanced motility (Fig 3) and resistance to fosfomycin (Fig 6). Notably, the Vfr effect on all these phenotypes was CavA-dependent. Moreover, the predicted T138 and T139 cAMP binding residues in the Vfr C-helix were essential for the Vfr functionality (Fig 3), however further biochemical assays are needed to fully elucidate the kinetics of this binding. Taken together, this verifies the link between the second messenger cAMP and the effector protein Vfr, and demonstrates that the cAMP-Vfr complex regulates *A*. *baumannii* biofilm, EPS production, motility and antibiotic resistance. However, it is unlikely to be the only cAMP effector encoded in the *A*. *baumannii* genome, future work will focus on identifying additional cAMP effectors initially exploring strains where *vfr* is absent (S2 Fig).

Surprisingly, the transcriptomics analysis (Fig 2A) revealed that cAMP lowers the expression of the QS autoinducer synthase coding gene *abaI*. Subsequent experiments validated that AHL production, and thus the QS system, in *A*. *baumannii* is inhibited by CavA (Fig 4). cAMP and QS interconnection has been demonstrated in other bacteria including the pathogens *V. cholerae*, *E*. *coli* and *P*. *aeruginosa* [32]. In *V*. *cholerae*, *V*. *vulnificus* and *E*. *coli*, cAMP-CRP mediates non-AHL dependent QS systems in opposite ways—in *V*. *cholerae* and *V*. *vulnificus*, cAMP stimulates production of autoinducer-2 (AI-2) and CAI-1 autoinducer respectively, whereas in *E*. *coli*, cAMP inhibits AI-2 production by decreasing the cognate synthase expression [32]. In contrast with this, cAMP was found to induce AHL-dependent QS system in *P*. *aeruginosa*, where the cAMP-Vfr complex stimulates the expression of *lasR* regulator [32]. Our results demonstrate a novel mechanism of cAMP-QS interplay in *A*. *baumannii* whereby cAMP inhibited the AHL-dependent QS system by reducing AbaI synthase expression and subsequently AHL production (Figs 2A and 4). Furthermore, decreased AbaI production has previously been linked to decreased *A*. *baumannii* virulence [17]. However, despite the decreased *abaI* transcription (Fig 2A), AB5075 is more virulent in high cAMP conditions (Fig 7). This indicates that cAMP modulates virulence in a QS-independent manner in *A*. *baumannii*.

In some Gram-negative bacteria the interplay between second messengers, such as cAMP and c-di-GMP, involves interactions between effector proteins [49]. Surprisingly, our data showed that CavA mediated transcriptional changes in c-di-GMP related genes via cAMP production in *A*. *baumannii* (Fig 2A). Intriguingly, the expression of one DGC and one PDE was increased (Fig 2A), suggesting that cAMP levels may be directly influencing c-d-GMP levels in this pathogen. In *P*. *aeruginosa*, c-di-GMP and cAMP levels are inversely proportional and the signalling cascades of the two second messengers are known to antagonistically regulate sessile and planktonic lifestyles [22,23]. Likewise, cAMP decreases sessility (Fig 1C), while c-di-GMP enhances biofilm formation [20] in *A*. *baumannii*, suggesting that the second messengers concentrations could be inversely proportional, following *P*. *aeruginosa* trends. Strikingly, we observed that decreasing cAMP concentrations led to a drop in c-di-GMP levels (Fig 5A). Moreover, we saw that this impact on c-di-GMP levels occurred via the cAMP-receptor protein Vfr (Fig 5B). Hence, in contrast to other bacteria where cAMP and c-di-GMP are inversely correlated [23], our findings indicate a novel interplay occurring in *A*. *baumannii* where the cAMP-Vfr complex stimulates c-di-GMP production at the global level. Further investigation of this interplay will uncover any feedback loops in this regulatory cascade.

A well-established paradigm in second messengers signalling is that high c-di-GMP concentrations promote high biofilm levels and low motility and vice versa [30]. *A*. *baumannii* is no exemption to this rule, as c-di-GMP signalling has been shown to induce sessility and inhibit motility [20]. Contrary to this, we observed that low cAMP concentrations or absence of Vfr increased biofilm formation and impaired motility (Figs 1C, 2C and 3), in spite of these conditions decreasing c-di-GMP levels (Fig 5). Hence, the c-di-GMP-biofilm paradigm is dependent on intracellular cAMP concentrations in *A*. *baumannii*, which clashes with the classical model depicting the regulation by these two molecules in other bacteria. However, our results could be attributed to compartmentalisation of c-di-GMP in the cells as a consequence of localised effect of DGCs and PDEs and the cognate c-di-GMP effectors. Nonetheless, we demonstrate a hierarchy in the singalling network of *A*. *baumannii*, atop of which is cAMP, which to the best of our knowledge is described for a first time.

**Fig 8:**
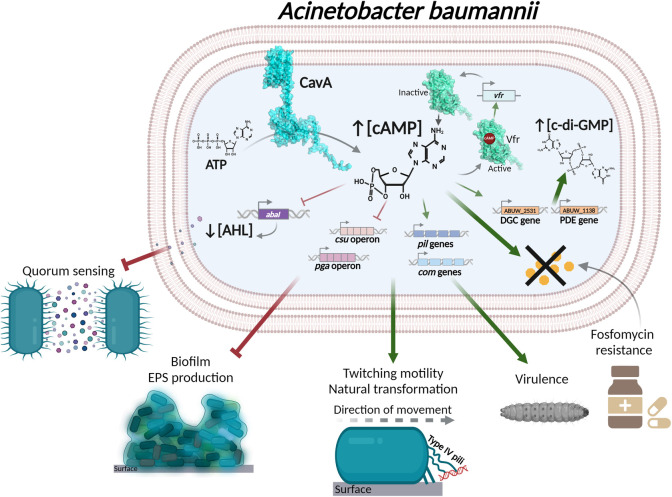
Model of the cAMP signalling cascade in *A. baumannii*. CavA adenylate cyclase catalyses the conversion of ATP to cAMP, increasing cAMP concentrations in the bacterial cells. Cyclic AMP then interacts with its effector protein Vfr, which autoregulates, triggering a positive feed-back loop. Increased cAMP concentrations decrease Csu pili genes and EPS producing machinery related genes (*pga*) expression, which leads to inhibition of biofilm formation. Meanwhile, cAMP increases the expression of type IV pili related genes (*pil* and *com*), resulting in increased bacterial motility and natural transformation, as well as virulence in *G. mellonella in vivo* infection model. High levels of cAMP also increase *A. baumannii* fosfomycin resistance. Moreover, cAMP affects quorum sensing by decreasing the expression of the autoinducer synthase gene *abaI*, which abolishes acyl-homoserine lactone (AHL) production and halts interbacterial signalling. Furthermore, cAMP increases the expression a diguanylate cyclase (DGC; synthesis of c-di-GMP) and a phosphodiesterase (PDE; degradation of c-di-GMP) gene expression, resulting in increased global c-di-GMP levels. Created using PyMOL and Biorender.com.

Overall, this study demonstrates the central role of cAMP-Vfr signalling in *A*. *baumannii* regulation of virulence related phenotypes including biofilm formation, motility and AMR (Fig 8). This work provides pioneering evidence that interbacterial communication and intracellular signalling in MDR *A*. *baumannii* are interconnected in a hierarchical network, atop of which is cAMP. These conclusions open the door to developing next-generation therapeutics targeting cAMP signalling, which would dismantle the central regulatory hub that controls important virulence factors of this critical-priority pathogen.

## Materials and methods

### Bacterial strains and growth conditions

*A*. *baumannii* AB5075-UW and its transposon mutants were obtained from the Manoil laboratory three-allele library [24]. Virulent opaque (VIR-O) variants of the wild-type and its derivative mutants were used due to their clinical relevance, unless otherwise stated [50]. Plasmids were stored in *E*. *coli* DH5α or DH5α λpir hosts when necessary. Cultures were grown routinely at 37°C shaking (180 rpm) or statically in LB broth or on LB agar respectively, unless stated otherwise. When required, the medium was supplemented with X-Gal (25 μg/ml), ampicillin (100 μg/ml), apramycin (100 μg/ml for *E*. *coli* and 200 μg/ml for *A*. *baumannii*), kanamycin (25 μg/ml), tellurite (6 μg/ml and 30 μg/ml for *E*. *coli* and *A*. *baumannii* respectively) and tetracycline (5 μg/ml). T26 transposon insertion in *vfr* transposon mutant AB07172 was validated using semi-degenerate PCR as described before [51]. Briefly, T26 fw1 oligo was used in combination with CEKG-2A, CEKG-2B and CEKG-2C for the first stage of the PCR. The PCR product was then amplified with T26 fw2/CEKG-4 primer pair in the second stage of the reaction. DNA fragments with different sizes were purified and sequenced using Sanger sequencing. The sequence of each fragment was aligned to the AB5075 genome to establish T26 transposon insertion position.

### Plasmid construction

All plasmids used in this work are listed in S1 Table. For clean deletion mutant construction, we followed the double-recombination strategy described by de Dios *et al*. involving the construction of pEMGT plasmid derivatives [26]. Approximately 1-kb regions flanking *cavA* (*ABUW_2208*), *cavB* (*ABUW_1085*) and *abaI* were amplified from genomic DNA (gDNA) of AB5075 using cavA up fw/rv and down fw/rv, cavB up fw/rv and down fw/rv and abaI up fw/rv and down fw/rv pairs of primers to amplify the upstream and downstream regions of each respective gene. The flanking regions of each gene were joined together via an overlapping PCR and cloned into pEMGT digested with SmaI, resulting in pEMGT-cavA, pEMGT-cavB and pEMGT-abaI.

For gene expression, a miniTn7 transposon derivative bearing the IPTG-inducible *lacI*^*q*^*-Ptac* expression system (pUC18T-miniTn7T-Tc-lacI^q^-Ptac [52], termed pJM101-Tc) was utilised. For similarly inducing gene expression in Tc-resistant genetic backgrounds (i.e. strains from the Manoil transposon mutant library), we generated a pUC18T-miniTn7T-Gm [53] (Addgene #63121) derivative bearing a tellurite resistance cassette. This cassette was obtained by cloning a fragment from pMo130-Tel [54] (Addgene #50799) cut with SmaI (containing the tellurite marker) into pUC18T-miniTn7T-Gm-lacI^q^-Ptac [55] digested with EagI and BsrGI and blunted with Klenow. This construction resulted in pUC18T-miniTn7T-Tel-lacI^q^-Ptac, termed PJM101-Tel.

The *cavA* gene was amplified from AB5075 gDNA using the cavA fw/rv oligos and digested with PstI and KpnI. The PCR product was then cloned in pUC18T-miniTn7T-Tc-lacI^q^-Ptac [52] digested with the same restriction enzymes (REs) resulting in pUC18T-miniTn7T-Tc-lacI^q^-Ptac::cavA.

To amplify the *vfr* (*ABUW_2741*) gene, the vfr fw RBS PstI/rv HindIII primer pair was used and the resulting product was digested with PstI and HindIII. It was then cloned in pUC18T-miniTn7T-Tel-lacI^q^-Ptac digested with the same REs. The derivative plasmid was pUC18T-miniTn7T-Tel-lacI^q^-Ptac::vfr. To generate a version of *vfr* carrying mutations in the cAMP binding site (T138A and T139W in the AB5075 *vfr* allele) as previously reported *in P*. *aeruginosa* [31], a joining PCRs were performed. The 5’ and 3’ regions of the *vfr* gene (approx. 0.4 kb each) were amplified using the vfr fw RBS PstI/vfr T138A T139W intra rv and vfr T138A T139W intra fw/vfr rv HindIII primer pairs, respectively, for introducing the T138A, T139W point mutations. The amplifications generated two overlapping fragments of the *vfr* gene containing the mutations. The overlapping fragment pair was assembled together by joining PCR, and the resulting mutated *vfr* gene fragment was digested with PstI and HindIII. This fragment was ligated into pUC18T-miniTn7T-Tel-lacI^q^-Ptac digested with the same enzymes, obtaining pUC18T-miniTn7T-Tel-lacI^q^-Ptac::*vfr*^T138A,T139W^.

For the construction of *Ppga* promoter fused to *gfpmut3*, 1-kb fragment including the promoter region was amplified via PCR using Ppga fw EcoRI/Ppga rv BamHI oligo pairs, digested with EcoRI and BamHI restriction enzymes and cloned in pUC18T-miniTn7T-zeo-gfpmut3 [56] (Addgene #65037) vector digested with the same enzymes. The obtained plasmid was pUC18T-miniTn7T-zeo-Ppga::gfpmut3. To assess *pilA* expression pUC18T-miniTn7T-zeo-PpilA::gfpmut3 [52] plasmid was used.

To increase the internal c-di-GMP levels in AB5075, pUC18T-miniTn7T-Tc-lacI^q^-Ptac and the heterologous DGC coding gene pleD* (constitutively active) [57] was generated. A DNA fragment containing the pleD* coding sequence plus a ribosome binding site was amplified from pMRB165 [58] with primers RBS fw PstI/pleD rv HindIII, digested with PstI and HindIII and cloned into pUC18T-miniTn7T-Tc-lacI^q^-Ptac digested with the same enzymes, resulting in pUC18T-miniTn7T-Tc-lacI^q^-Ptac::pleD*. To decrease c-di-GMP levels similarly, a pUC18T-miniTn7T-Tc-lacI^q^-Ptac derivative bearing the PDE coding gene *rocR* [59] was generated. The *rocR* coding sequence was amplified from *P*. *aeruginosa* DSM 50071T [60] using primers rocR RBS fw HindIII/rocR rv (the same ribosome binding site as for the pleD* construction was used by including it in the forward primer), digested with HindIII and cloned into pUC18T-miniTn7T-Tc-lacI^q^-Ptac cut with HindIII and NruI, resulting in pUC18T-miniTn7T-Tc-lacI^q^-Ptac::rocR.

To generate a pWH1266 [61] derivative that could be selected in MDR AB5075 and its derivative transposon mutants from the Manoil library, an apramycin resistance marker was PCR amplified from pFLAG-attP (Addgene, #110095) using primers Apr fw/Apr rv [26] and cloned into pWH1266 digested with ScaI, resulting in pWH1266-Apr.

To construct a variant of the CensYBL c-di-GMP biosensor [35] within an *A*. *baumannii* replicative vector, a multi-step cloning strategy was followed. First, a DNA fragment containing the *tetR-tetP* expression system plus a ribosome binding site was amplified from pYDE009 [62] with primers tetR RBS fw NdeI/tetR rv NruI and ligated into pBluescript-II-SK(+) (pBSK) cut with EcoRV. The orientation of the fragment was assessed to select a clone bearing the *tetP* promoter neighbouring the HindIII restriction site in the cloning vector, whereas the 3’ end of *tetR* would be neighbouring the SmaI site. This intermediate vector was digested with HindIII and NdeI, generating a 0.69 Kb fragment containing a truncated segment of the *tetR-tetP* system plus the rest of the vector backbone. On the other hand, the pCensYBL plasmid was digested with HindIII and NdeI, generating a 2.38 Kb fragment including the promoter-less CensYBL construct. The truncated *tetR-tetP* fragment, the CensYBL construct and the adapted pBSK vector backbone were ligated together, generating a pBSK-tetR-CensYBL intermediate. This plasmid was digested with SmaI and SalI, and the resulting 3.14 Kb fragment containing the tetR-CensYBL construct was cloned into pWH1266-Apr digested with EcoRV and SalI, resulting in pCensYBL-Ab. An inactive version of this biosensor (pCensYBL*-Ab) was generated as a control, following the same cloning strategy and using pCensYBL* (inactive version of the biosensor) [35] as initial material.

All constructs were verified by Sanger sequencing.

### Strain construction

A list of all strains used in this work is given in S1 Table. AB5075 derivatives with in-frame gene deletion were created using our previously described *A*. *baumannii* genome-editing toolkit [26]. Briefly, triparental mating was used to transfer pEMGT derivatives (pEMGT-cavA, pEMGT-cavB and pEMGT-abaI) from DH5α λpir into the parental AB5075 strain using pRK2013 as helper plasmid. After selecting the first recombination event, the second event leading to the gene deletion was triggered by conjugating pSW-Apr into the cointegrate strains [26,63]. This created Δ*cavA*, Δ*cavB*, Δ*cavA*Δ*cavB*, Δ*abaI*, Δ*cavA*Δ*abaI*, Δc*avA*/*csuC*::Tn and Δc*avA*/*pgaA*::Tn strains respectively. Deletions were verified by PCR.

For complementation, previously described four-parental mating method [64] with pRK2013 and pTNS2 helper plasmids [63,65] was used. Derivatives of miniTn7T-Tc [52] and miniTn7T-Tel bearing the gene of interest or empty were inserted in the chromosome of intended strains. Clones were picked on selective medium depending on the resistance marker in the miniTn7T and insertions were verified using AB5075-glmS fw/Tn7R primers [52,66].

To generate AB5075 derivatives carrying *gfp* transcriptional fusions to the *pilA* and *pga* promoters, pUC18T-miniTn7T-zeo-PpilA::gfpmut3 and pUC18T-miniTn7T-zeo-Ppga::gfpmut3 were introduced in AB5075 by three-parental mating and their integration in the chromosome by single recombination was selected. Selection was performed on LB agar supplemented with gentamycin (20 mg/L) and zeocin (500 mg/L). Insertions were validated by PCR using PpilA fw EcoRI/miniTXC gfp rv Ppga fw EcoRI/miniTXC gfp rv primer pairs.

### Protein sequence analysis

Amino acid sequences for CavA (AKA31936.1) and CavB (AKA30837.1) were obtained from the *A*. *baumannii* AB5075-UW genome (CP008706.1). Proteins domains and cellular localisation were predicted with InterProScan and TMHMM (v2.0) softwares [67]. IBS 2.0 [68] and BioRender.com were used to create figures. These proteins were searched in a pangenome of 9,696 *A*. *baumannii* genomes [69], from which their isolation site was taken and classified into 4 groups: Human, Hospital environment, Environmental, and Other. Protein structures were predicted using AlphaFold [70,71] and visualised with PyMOL (v3.0) software, where “cealign” command was used for protein structures alignment.

### Biofilm formation assay

To examine regulators of biofilm formation of AB5075, all biofilm formation assays including the initial screen were performed following a previously described protocol [72] with slight changes. Overnight cultures were diluted to OD_600_ 0.1 and 150 μl were used to inoculate the corresponding wells of a microtiter dish. Following 24 h incubation at 37°C shaking (180 rpm) and growth assessment at OD_600_, planktonic cells were removed by washing the wells three times with distilled water. Biofilms were then stained with 200 μl 0.1% Crystal violet for 15min and the excess stain was removed by five consecutive washings with distilled water. Plates were left to air-dry and biofilm-bound stain was resolubilised in 200 μl 99% Ethanol. Absorbance was measured at 570 nm (OD_570_) using SPECTROstar plate reader (BMG Labtech). The results shown are the average of three biological replicates, with three technical replicates each.

### Intracellular cAMP quantification

Intracellular cAMP levels were measured as described before [18] with slight modifications. Bacterial cultures grown to OD_600_ 0.7 were used to harvest cells via centrifugation at 5,000 rpm and 4°C for 20 min. Cells were resuspended in 3ml PBS and lysed by sonication for 5 min (5 sec on, 15 sec off) at 50% amplitude. Samples were centrifuged again as already described to remove cell debris and collect the supernatant. Coomassie (Bradford) Protein Assay kit (23200) was used to quantify the protein concentration of each sample. Briefly, 250 μl Coomassie reagent were added to 5 μl sample or BSA standard, mixed briefly on a plate shaker and incubated at room temperature for 10min. Absorbance was measured at 595 nm and protein concentrations were calculated from a standard curve. Then Cyclic Nucleotide XP Enzymatic Immunoassay (Cell Signalling Technology, MA, USA) was used following manufacturer’s instructions to quantify cAMP concentrations. Fifty microlitres of each sample were transferred in duplicates in the microwell strips provided and 50 μl horseradish peroxidase-linked cAMP were added. Strips were left for 3 h at room temperature on a plate shaker. Liquid was removed from the strips and each well was washed four times with 200 μl wash buffer. After that 100 μl of stop solution (3,3’,5,5’-tetramethylbenzidinesubstrate) were added and absorbance was measured at 490 nm. Cyclic AMP concentrations were calculated from the inverse for the standards from a standard curve and adjusted for the protein concentration of each sample. The data presents three biological repeats with two technical in each.

### Differential RNA sequencing and transcriptomic analysis

Cultures of Δ*cavA* EV and Δ*cavA*+*cavA* were grown in LB broth with IPTG (1 mM) to mid-log phase (OD_600_ 0.7) at 37°C, 180 rpm. Cells were pelleted by centrifugation and resuspended in RNAlater. RNAeasy kit with on-column DNase digestion (Quiagen) was used for total RNA isolation. RNA quantity and quality were assessed using Agilent RNA 6000 Nano Kit and Agilent 2100 Bioanalyzer according to the total RNA concentration and the rRNA peak profile and sharpness obtained for each sample. Samples were sequenced using Illumina NovaSeq X Plus with 12 million reads per sample at Microbial Genome Sequencing Centre (Pittsburgh, Pennsylvania, U.S.A). Demultiplexing, quality control, and adapter trimming was done with bcl-convert (v4.1.5). BioJupies [73] was used to create a volcano plot. Genes were defined as differentially expressed if Log2(fold change) ≥ 1 or ≤ -1 and p value < 0.05. FUNAGE-Pro [27] with preset settings was used for Gene Set Enrichment Analysis (GSEA).

The RNA sequencing datasets are available at the Gene Expression Omnibus repository (NCBI) under the accession number GSE250425.

### Twitching motility assay

For assessing CavA and Vfr roles in the motility regulation, previously described twitching assay was used [52]. AB5075 and its derivative strains were grown overnight on LB agar. Following autoclaving, twitching medium (10 g Tryptone, 5 g Yeast extract, 10 g Agar per 1 L) was supplemented with IPTG (1 mM) when needed, 10 ml were poured per Petri dish and plates were dried aseptically for 8 min. Each plate was inoculated at the plastic-medium interface by piercing a single colony from the plate culture through the agar. All plates were incubated at 37°C. After 48h the agar was gently removed and the diameter of the movement trace was measured. The experiment was repeated three independent times.

### Natural transformation assay

Natural transformation assays were performed following a modification of our previously published protocol [52]. Overnight cultures of *A*. *baumannii* AB5075 and the Δ*cavA* mutant were diluted 1:100 (v/v) in 5 ml of LB broth supplemented with CaCl_2_ 2 mM. Cultures were incubated at 37°C, 180 rpm until reaching OD_600_ 0.8. 20 μl of each bacterial culture were mixed with 1 μg of purified genomic DNA from an AB5075 derivative carrying a chromosomal miniTn7T-Tc transposon insertion with a tetracycline resistance cassette (AB5075/miniTn7T-Tc-lacI^q^-Ptac). The mixtures were spotted on twitching agar (see twitching motility assay procedure) supplemented with CaCl_2_ 2 mM. The mixtures were left to air-dry in a laminar flow hood and were incubated for 4 h at 37°C. After this, the biomass was resuspended in 1 ml of PBS buffer and serial dilutions were plated either on selective LB agar supplemented with tetracycline 5 mg/L or on plain LB agar to assess viability. The transformation frequency was calculated as the number of transformant cells per millilitre divided by the number of viable cells per millilitre. Four biological replicates were performed for each strain.

### GFP-based transcriptional fusions assay

The expression from the *PpilA* and *Ppga* promoters was measured following a previously described protocol [53] with slight changes. AB5075 WT, Δ*cavA* and *vfr*::Tn strains bearing mini-Tn7T-based insertions with the *PpilA*::*gfpmut3* and *Ppga*::*gfpmut3* transcriptional fusions were used. Cultures, grown overnight, were diluted 1:100 in LB broth and grown for 4.5 h at 37°C shaking (180rpm). Cells were harvested via centrifugation, resuspended in PBS and 100 μl were transferred in triplicates in a 96-well plate. Samples growth (OD_600_) and GFP fluorescence (excitation: 485 nm; emission: 535 nm) were measured. The fluorescence was then normalised to the respective growth measurement. The experiment was repeated three independent times.

### Congo red exopolysaccharide assay

CavA and Vfr effect on EPS production was tested by adapting a protocol from [74]. Adjusted overnight cultures to OD_600_ 1 were used to inoculate 1% Tryptone with 1% Agar containing Congo red (40 μg/ml) and Coomassie brilliant blue (20 μg/ml) dyes. Plates were incubated at 37°C for 5 days. Example images of the colonies from three independent repeats are presented.

### Detection of exogenous acyl-homoserine lactones (AHL)

To assess the effect of CavA on QS, a previously described method for detecting AHLs in *A*. *baumannii* [75] was used with some modifications. Briefly, *A. tumefaciens traG*::*lacZ* biosensor strain grown to OD_600_ 0.3 was mixed with X-Gal (75 μg/ml) and soft agar (LB 0.7% agar). Plates were dried at 37°C for 35 min. Cultures of avirulent translucent (AV-T) AB5075 and its derivatives were adjusted to OD_600_ 0.3 and 1 μl was spot plated in triplicates on the soft agar. Plates were incubated at 28°C for approximately 36 h. The diameter of the blue halos indicating production of AHL was measured. The experiment was done in three biological repeats.

### Quantification of c-di-GMP levels

Cyclic di-GMP synthesis was measured as already described [35] with some changes. Overnight cultures of strains with active (CensYBL-Ab) and inactive (CensYBL*-Ab) c-di-GMP biosensor were diluted 1:100 in fresh LB broth and incubated at 37°C shaking for 1 h. Following biosensor expression induction with anhydrotetracycline (50 ng/ml), strains were incubated for further 2 h. Cells harvested via centrifugation from 300 μl of each culture were resuspended in 1 ml sterile PBS. 100 μl/well in triplicates were loaded in a dark 96-well plate. Fluorescence was measured using CLARIOstar plate reader (BMG Labtech). mCherry and YFP were detected using excitation/emission wavelengths of 570-15/620-20 nm and 497-15/540-20 nm respectively. Cyclic-di-GMP levels were calculated by normalising the YFP to the mCherry fluorescence data. The averages of three biological repeats with three technical replicates are presented.

### Virulence in *G. mellonella in vivo* model

Virulence assays were performed using healthy *G*. *mellonella* (UK Waxworms Ltd.) larvae of similar size. *A*. *baumannii* AB5075 or mutant derivative cells were diluted to OD_600_ 1.0 and serially diluted in PBS. The number of viable cells (enumerated by plating the serial dilutions on LB agar) of each injected strain per replicate are listed in S2 Table. All larvae were left at 37°C for 48 h. *G*. *mellonella* survival was assessed every 2 h between 20–30 h interval post-injection and at 48 h post-infection. Ten larvae were injected in each biological replicate and the experiment was repeated three independent times. Death was assessed by complete lack of response to mechanical stimuli. Probability of survival was assessed using the Long-rank (Mantel-Cox) method on GraphPad Prism.

### Antibiotic disc diffusion assay

Cation-adjusted Mueller-Hinton (CAMH) agar (pH 7.4, CaCl_2_ 2 mM, MgSO_4_ 1mM) (Sigma-Aldrich) was used to assess antibiotic susceptibility. Overnight cultures of *A*. *baumannii* AB5075 and its derivatives were adjusted to OD_600_ 0.1 in LB broth. Sterile cotton swabs were used to spread the adjusted bacterial cultures and discs with 50 μg fosfomycin were placed in the centre of each plate. All plates were left at 37°C for 24 h. The experiment was repeated three independent times.

### Statistics

Graphs represent average ± standard deviation (SD) of the data. All statistical tests were performed on GraphPad Prism 10.1.0 (316) software (GraphPad Software, San Diego, California USA, www.graphpad.com). One-way ANOVA with Dunnett and Tukey’s post-hoc tests or Student t-test were used when indicated to analyse the data. *G*. *mellonella* survival probability was analysed using Long-rank (Mantel-Cox) test.

## Supporting information

S1 Fig Validation of *ABUW_2208* (*cavA*) transposon mutants as well as schematic representation of CavA and CavB domains, and their mutants effect on growth, biofilm formation and cAMP concentrations in *A*. *baumannii* AB5075. **A**–PCR validating T26 insertion in the *ABUW_2208* gene in five strains annotated as *ABUW_2208*::T26 transposon mutants, labelled with their corresponding number from the Manoil transposon mutant library. C.*ABUW_2208* fw/rv (annealing at the beginning and end of the *ABUW_2208* gene) were used. Wild-type *ABUW_2208* gene is 1.5-kb in size (C+), while the *cavA* gene with T26 transposon (1.8-kb) is ~3.2-kb. Genomic DNA (20 ng) from AB5075 was used as positive control (C+) and water was used as a negative one (C-). Only AB05781, AB05784 and AB05783 were confirmed to be *ABUW_2208*::T26 mutants. The other two strains (AB05780 and AB05782) did not harbour the T26 transposon in the *ABUW_2208* gene and thus, were incorrectly assigned as *ABUW_2208*::T26 mutants. **B**–Schematic representation of CavA protein (489 aa) showing its predicted transmembrane (TM) regions and the adenylate/guanylate cyclase domain (PF00211). **C & D**–Growth measured as optical density at 600 nm (OD_600_) (**C**) and biofilm formation measured as optical density at 570 nm (OD_570_) (**D**) after 24 h at 37°C shaking, demonstrating that both phenotypes remained unchanged in the controls bearing chromosomal insertion of the empty miniTn7 (EV) in the wild-type (WT) and Δ*cavA* backgrounds. **E**—Intracellular cAMP concentrations presented as μmol per milligram protein of wild-type (WT), Δ*cavA* and Δ*cavB* single and Δ*cavA*Δ*cavB* double mutants and their derivatives with EV showing that the empty miniTn7 did not alter cAMP production in these strains. ns p>0.05, *p<0.05, **p<0.01, ****p<0.0001 One-Way ANOVA with Tukey post-hoc test. **F**–Representation of CavB protein (487 aa) with its CYTH (PF01928) and CHAD (PF05235) domains. T26 transposon insertion positions in the transposon mutants from the Manoil mutant library [24] are presented by red triangles. **G**–Growth (OD_600_) of Δ*cavB* mutant and WT after 24 h period incubation at 37°C shaking. ns p>0.05—Unpaired t-test.
(TIF)

S2 Fig Conservation of CavA, CavB and Vfr across the *A*. *baumannii* pangenome. **A**—Heatmap of the protein profile of CavA, CavB and Vfr in the *A*. *baumannii* pangenome. The genomes are clustered (rows) by the variants of the proteins they present. The most frequent variant of the protein (ref) is depicted in orange and the other variants are shown in different shades of purple. The left side of the heatmap shows the metadata: Multilocus sequence typing (MLST) (the 5 most frequent, highlighting ST2), and host source (predominantly human but also hospital environment, environment and other). Highlighted are *A*. *baumannii* strains commonly used as reference: ATCC17978 (GCF_902728005.1), ATCC19606 (GCF_014116795.1), ACICU (GCF_000018445.1), AYE (GCF_000069245.1), AB5075 (GCF_000770605.1), AB0057 (GCF_000021245.2). **B**—Proportion of different sequence variants (v) of CavA, CavB and Vfr proteins in the *A*. *baumannii* pangenome. The vref variant represents the most frequent, and "not found" appears when the protein has not been found. The number of genomes per group is as follows: Human (6589), Hospital environment (131), Environmental (140), Other (2836).
(TIF)

S3 Fig CavA regulates *A*. *baumannii* EPS production is via PNAG production modulation and its regulation of biofilm formation is multifactorial. **A**–Representative images of Congo agar plates showing that the empty miniTn7 vector does not affect *A*. *baumannii* EPS production. B–Representative image of Congo red plates demonstrating the effect of *pgaA* disruption (*pgaA*::Tn) on Congo red dye binding to *A*. *baumannii* EPS and on the increased EPS production in the Δ*cavA* mutant. This shows that the effect of *cavA* on EPS production is mainly due to changes in the production of poly-β-1,6-N-acetylglucosamine (PNAG) and *pgaABCD* operon expression. **C** & **D**–Growth (**C**) and biofilm formation (**D**) of AB5075 WT and its derivative Δ*abaI*, *csuC*::Tn, *pgaA*::Tn and Δ*cavA* single and double mutants after being incubated at 37°C shaking for 24 h. Deletion of the autoinducer synthase gene *abaI* in Δ*cavA* did not affect the increased Δ*cavA* biofilm. In contrast, disruption of *csuC* or *pgaA* in the Δ*cavA* mutant significantly decreased the high biofilm levels caused by the deletion of *cavA* but did not completely reversed the Δ*cavA* phenotype. This data demonstrates that the regulation of *A*. *baumannii* biofilm formation by CavA is dependent on Csu pili and EPS production and is the additive effect of the global simultaneous regulation of multiple genes. ns p>0.05, **p<0.01, **** p<0.0001—One-Way ANOVA with Tukey post-hoc test.
(TIF)

S4 Fig Dataset with controls for twitching motility and CavA effect on natural transformation. **A**–Twitching motility of Δ*cavA* related strains showing that miniTn7 EV has no impact on *A*. *baumannii* motility. **B**–Natural transformation of Δ*cavA* mutant was bellow the detection limit (< d. l.) and was significantly decreased compared to the WT AB5075. ns p>0.05, ** p<0.01, ***p<0.001, **** p<0.0001—One-Way ANOVA with Tukey post-hoc test (**A**) and Unpaired t-test (**B**).
(TIF)

S5 Fig Vfr structure analysis. **A**—Protein sequence alignment comparing Vfr from *A. baumannii* AB5075 and its orthologue from *P. aeruginosa* PAO1. Residues involved in the interaction with cAMP, according to Beatson *et al*. [76], are framed in red. The strong conservation between these residues in both orthologues suggests that both proteins will interact with cAMP with the same specificity. The DNA binding domain of both proteins, as predicted by an InterPro sequence scan (PF00325), appears framed in green. The conservation in this region suggests a similar target promoter sequence for both orthologues. (*): identical residues; (:): highly similar residues; (.): somewhat similar residues. The sequence alignment was performed using Clustal Omega. **B-D–**Structural alignment of Vfr^AB5075^ and Vfr^PAO1^ in PyMOL. Overall alignment of the two proteins (Vfr^AB5075^ in magenta and Vfr^PAO1^ in cyan) indicates root mean square deviation (RMSD) of 1.938811 over 208 residues (**B**). Highlighted are the DNA binding domains (**C**) and cAMP binding sites (**D**) in Vfr^AB5075^ (magenta) and Vfr^PAO1^ (blue). **E**–Two residues (threonine 138 and threonine 139) from the Vfr^AB5075^ cAMP binding site (magenta) were modified to alanine and tryptophan respectively resulting in Vfr^T138A,T139W^ variant which was subsequently used to demonstrate the necessity of cAMP binding for proper Vfr function in *A*. *baumannii*. Protein structure visualised using PyMOL.
(TIF)

S6 Fig Datasets including controls for Vfr effect on growth, biofilm, EPS and motility of *A*. *baumannii*. Growth (**A**), biofilm formation (**B**), EPS production (**C**) and motility (**D**) of *cavA* and *vfr* related strains demonstrating that the observed phenotypes were not attributed to growth alternations of the strains. Moreover, the empty miniTn7 system (EV) with Tetracycline (Tc) or Tellurate (Tel) resistance markers, used for the complementations and genes expression in different backgrounds, did not have an effect on any of the tested phenotypes. Growth and biofilm formation were assessed after 24 h at 37°C shaking. EPS production was assessed on Congo agar after 5 days incubation of the strains at 37°C and motility was tested after 48 h on soft agar at 37°C. ns p>0.05, *p<0.05, ** p<0.01, *** p<0.001, **** p<0.0001—One-Way ANOVA with Tukey post-hoc test.
(TIF)

S7 Fig *PpilA* and *Ppga* transcriptional regulation by CavA and Vfr in *A*. *baumannii*. Expression of *pilA* gene (A) and *pga* operon (B) is regulated by CavA and Vfr. Promoter regions of *pilA* and *pga* fused with *gfpmut3* fluorescent reporter (*PpilA*::*gfpmut3* and *Ppga*::*gfpmut3* respectively) were used to assess the effect of CavA and Vfr on their expression. Bacterial cells were harvested from diluted bacterial cultures grown for 4.5 h, after which were resuspended in sterile PBS. Fluorescence (A.U.) at 470-15/515-20 nm excitation/emission was measured to determine the expression of Gfp and thus the expression of each promoter. Optical density at 600 nm (OD_600_) was measured to determine growth. Data represents the average of three independent repeats ± SD. **p<0.01, ****p<0.0001 –One-Way ANOVA with Tukey post-hoc test.
(TIF)

S8 Fig Representative image of a plate with AHL biosensor including *A*. *baumannii* control strains. AHL production (indicated by the presence of a blue halo around the colonies) by the AV-T variants of the wild-type (WT) AB5075 and its *cavA* related derivatives. Dramatic difference was observed in the AHL synthesis, as the deleted Δ*cavA* mutant had increased AHL production compared to the WT and complemented Δ*cavA*+*cavA* strains where AHL secretion was abolished. AHL production was unaffected by the chromosomal insertion of the empty miniTn7 vector (EV) in the WT and Δ*cavA* backgrounds.
(TIF)

S9 Fig Cyclic di-GMP biosensor. **A—**Cyclic di-GMP levels in WT AB5075, WT overexpressing constitutively active DGC gene pleD* (WT+pleD*) and WT with empty miniTn7 used for the strain construction. This demonstrates the activity of the modified CensYBL-Ab in detecting the elevated c-di-GMP levels in the WT+pleD* strain. The inactive CensYBL*-Ab biosensor was used to demonstrate that the increase in the signal was due to the changing c-di-GMP levels. **B**–Cyclic di-GMP levels in *cavA* related strains compared to the parental WT AB5075. The empty miniTn7 was used as control which demonstrates the empty vector did not have an effect on the c-di-GMP levels in the WT or the deleted Δ*cavA* mutant. ns p>0.05, ** p<0.01, **** p<0.0001—Two-Way ANOVA (**A**) and One-Way ANOVA (**B**) with Tukey post-hoc test.
(TIF)

S10 Fig Disc diffusion assays for testing the role of CavA and Vfr in *A*. *baumannii* antibiotic resistance. **A**–WT AB5075 and Δ*cavA* resistance to different classes of antibiotics such as quinolones (ciprofloxacin, 5 μg), polymyxins (colistin, 10 μg), amphenicols (chloramphenicol, 50 μg) and aminoglycosides (tobramycin, 30 μg). **B**—Full dataset of WT and its *cavA* and *vfr* related derivatives to fosfomycin (50 μg). Strains harbouring empty miniTn7 (EV) with tetracycline (Tc) or tellurate (Tel) resistance cassette were used as controls demonstrating that the resistance phenotype was unaffected by the presence of the empty vectors. ns p>0.05, *p<0.05, ** p<0.01, *** p<0.001, **** p<0.0001 –Two-Way ANOVA with Sidak post-hoc test (**A**) and One-Way ANOVA with Tukey post-hoc test (**B**).
(TIF)

S1 Table Strains, plasmids and primers used in this work. (XLSX)

S2 Table Viable cells injected per *Galleria mellonella* larvae in each of the *in vivo* virulence assay replicates for each strain. Colony forming units were enumerated by serial dilution and plating on LB agar using the same cell suspensions that were used for injection.
(XLSX)

S3 Table dRNA-seq data of differentially expressed genes in Δ*cavA*+*cavA* complemented strain vs the control Δ*cavA* EV strain. (XLSX)

S4 Table Gene set enrichment analysis results of the significantly regulated genes in Δ*cavA+cavA* complemented vs Δ*cavA* EV strain in the dRNA-seq experiment. Results were obtained using FUNAGE-Pro.
(XLSX)
