# Supplementary figures and images for: Cyclic AMP is a global virulence regulator governing inter and intrabacterial signalling in Acinetobacter baumannii
Source: PLoS Pathog. 2024 Sep 6;20(9):e1012529. doi: 10.1371/journal.ppat.1012529 (PMC11410210; doi:10.1371/journal.ppat.1012529)

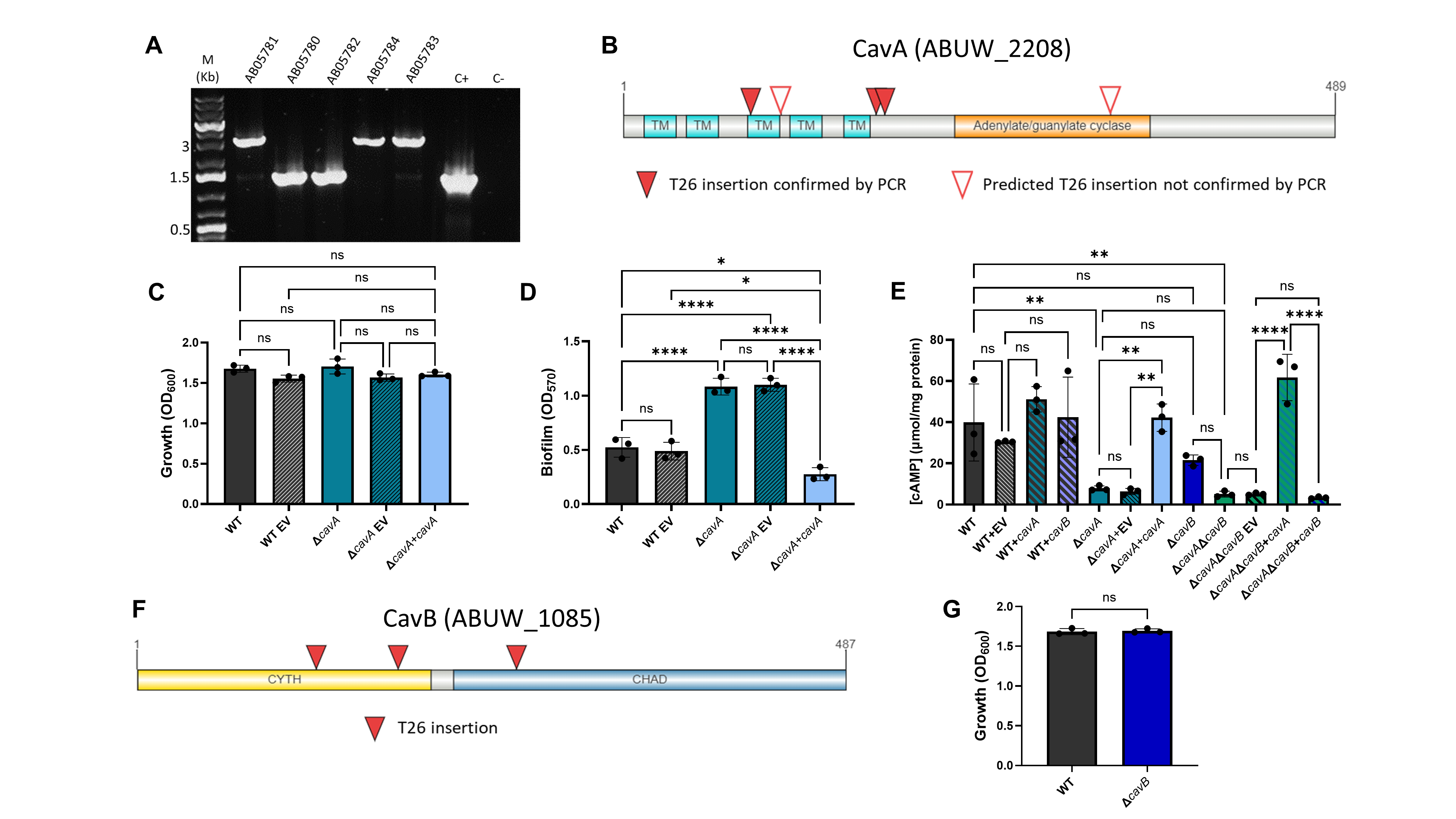

Supplement: S1 Fig — A–PCR validating T26 insertion in the ABUW_2208 gene in five strains annotated as ABUW_2208::T26 transposon mutants, labelled with their corresponding number from the Manoil transposon mutant library. C.ABUW_2208 fw/rv (annealing at the beginning and end of the ABUW_2208 gene) were used. Wild-type ABUW_2208 gene is 1.5-kb in size (C+), while the cavA gene with T26 transposon (1.8-kb) is ~3.2-kb. Genomic DNA (20 ng) from AB5075 was used as positive control (C+) and water was used as a negative one (C-). Only AB05781, AB05784 and AB05783 were confirmed to be ABUW_2208::T26 mutants. The other two strains (AB05780 and AB05782) did not harbour the T26 transposon in the ABUW_2208 gene and thus, were incorrectly assigned as ABUW_2208::T26 mutants. B–Schematic representation of CavA protein (489 aa) showing its predicted transmembrane (TM) regions and the adenylate/guanylate cyclase domain (PF00211). C & D–Growth measured as optical density at 600 nm (OD600) (C) and biofilm formation measured as optical density at 570 nm (OD570) (D) after 24 h at 37°C shaking, demonstrating that both phenotypes remained unchanged in the controls bearing chromosomal insertion of the empty miniTn7 (EV) in the wild-type (WT) and ΔcavA backgrounds. E—Intracellular cAMP concentrations presented as μmol per milligram protein of wild-type (WT), ΔcavA and ΔcavB single and ΔcavAΔcavB double mutants and their derivatives with EV showing that the empty miniTn7 did not alter cAMP production in these strains. ns p>0.05, *p<0.05, **p<0.01, ****p<0.0001 One-Way ANOVA with Tukey post-hoc test. F–Representation of CavB protein (487 aa) with its CYTH (PF01928) and CHAD (PF05235) domains. T26 transposon insertion positions in the transposon mutants from the Manoil mutant library [24] are presented by red triangles. G–Growth (OD600) of ΔcavB mutant and WT after 24 h period incubation at 37°C shaking. ns p>0.05—Unpaired t-test. (TIF) [file ppat.1012529.s001.TIF]

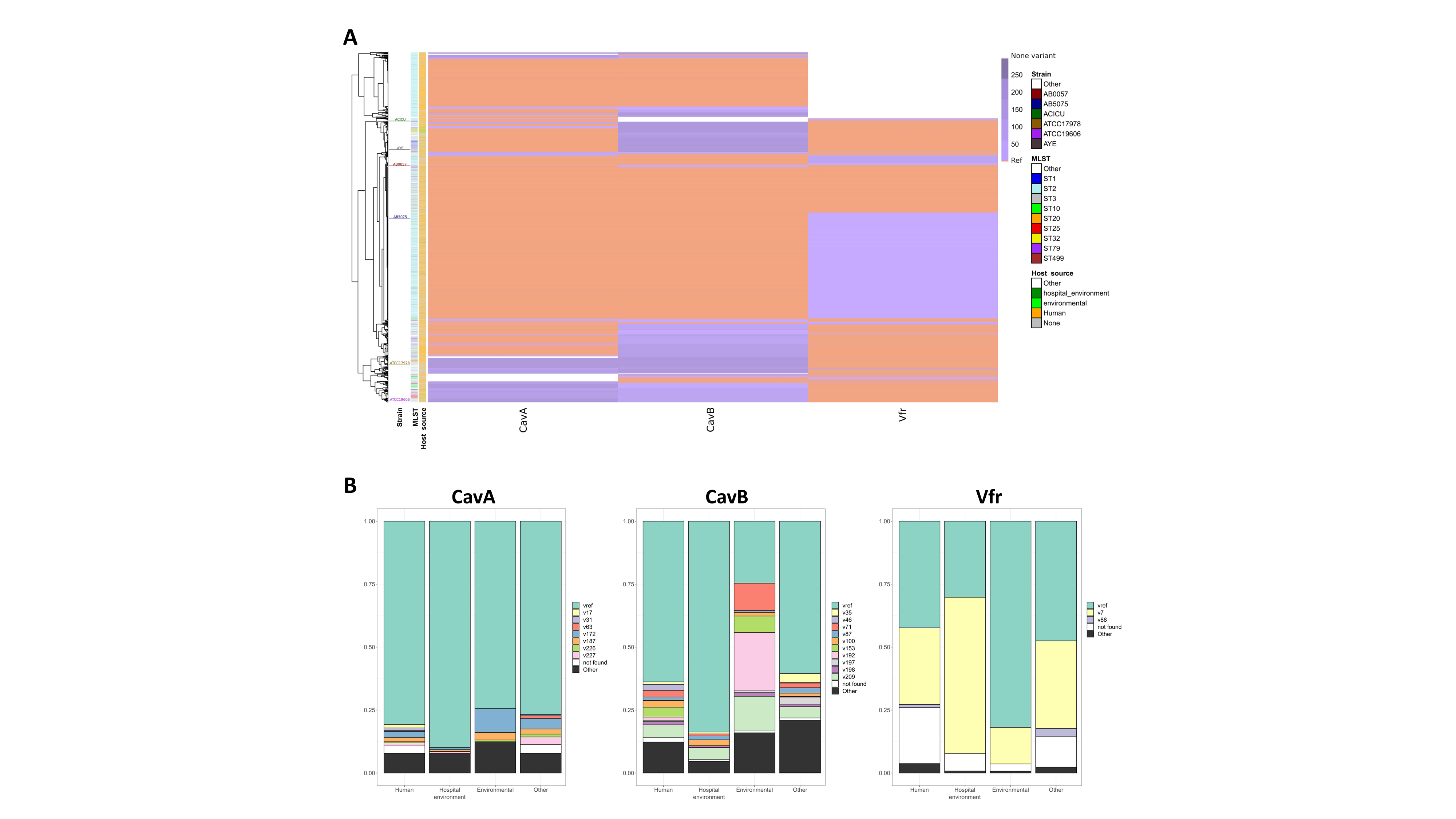

Supplement: S2 Fig — A—Heatmap of the protein profile of CavA, CavB and Vfr in the A. baumannii pangenome. The genomes are clustered (rows) by the variants of the proteins they present. The most frequent variant of the protein (ref) is depicted in orange and the other variants are shown in different shades of purple. The left side of the heatmap shows the metadata: Multilocus sequence typing (MLST) (the 5 most frequent, highlighting ST2), and host source (predominantly human but also hospital environment, environment and other). Highlighted are A. baumannii strains commonly used as reference: ATCC17978 (GCF_902728005.1), ATCC19606 (GCF_014116795.1), ACICU (GCF_000018445.1), AYE (GCF_000069245.1), AB5075 (GCF_000770605.1), AB0057 (GCF_000021245.2). B—Proportion of different sequence variants (v) of CavA, CavB and Vfr proteins in the A. baumannii pangenome. The vref variant represents the most frequent, and "not found" appears when the protein has not been found. The number of genomes per group is as follows: Human (6589), Hospital environment (131), Environmental (140), Other (2836). (TIF) [file ppat.1012529.s002.TIF]

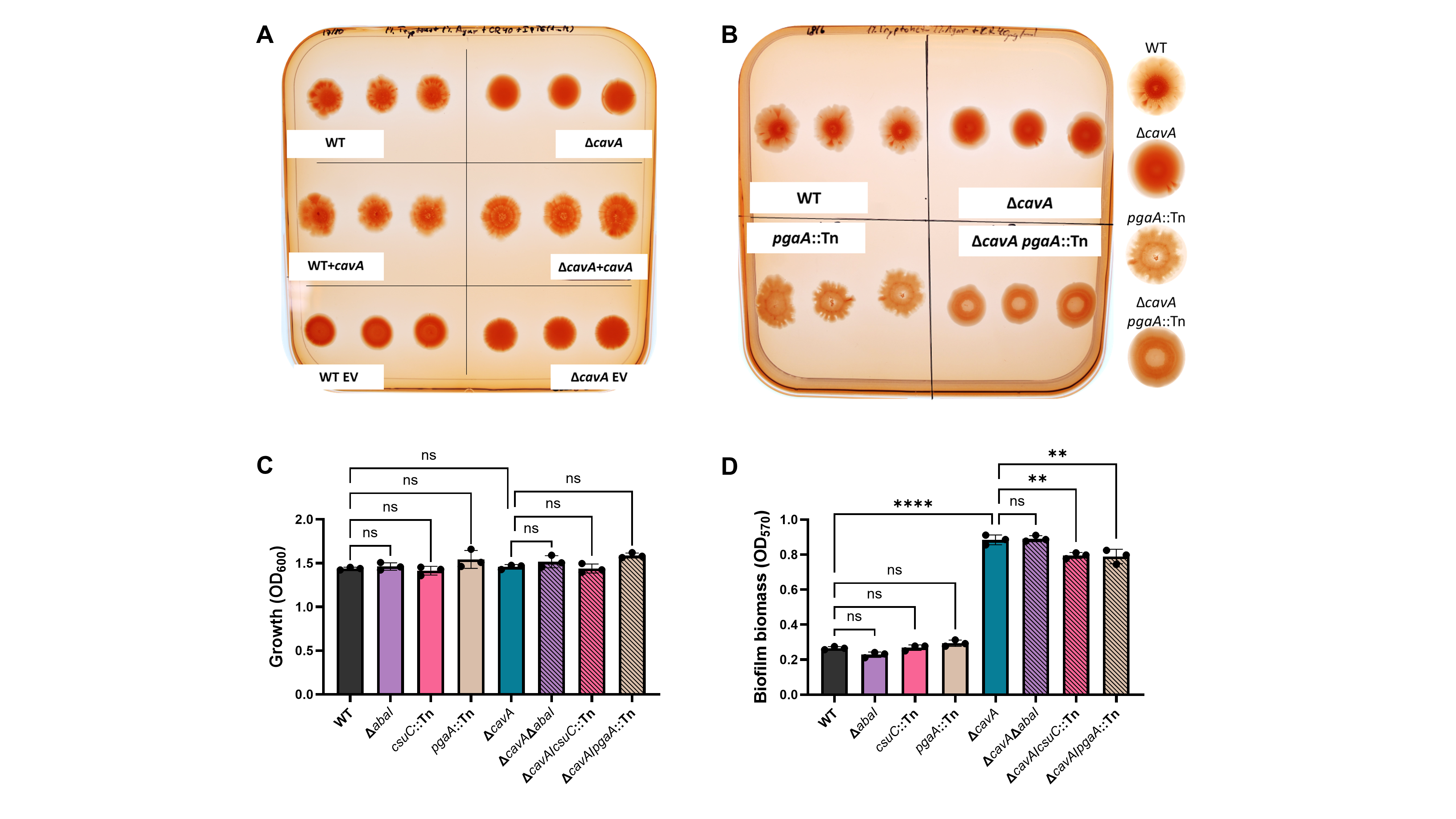

Supplement: S3 Fig — A–Representative images of Congo agar plates showing that the empty miniTn7 vector does not affect A. baumannii EPS production. B–Representative image of Congo red plates demonstrating the effect of pgaA disruption (pgaA::Tn) on Congo red dye binding to A. baumannii EPS and on the increased EPS production in the ΔcavA mutant. This shows that the effect of cavA on EPS production is mainly due to changes in the production of poly-β-1,6-N-acetylglucosamine (PNAG) and pgaABCD operon expression. C & D–Growth (C) and biofilm formation (D) of AB5075 WT and its derivative ΔabaI, csuC::Tn, pgaA::Tn and ΔcavA single and double mutants after being incubated at 37°C shaking for 24 h. Deletion of the autoinducer synthase gene abaI in ΔcavA did not affect the increased ΔcavA biofilm. In contrast, disruption of csuC or pgaA in the ΔcavA mutant significantly decreased the high biofilm levels caused by the deletion of cavA but did not completely reversed the ΔcavA phenotype. This data demonstrates that the regulation of A. baumannii biofilm formation by CavA is dependent on Csu pili and EPS production and is the additive effect of the global simultaneous regulation of multiple genes. ns p>0.05, **p<0.01, **** p<0.0001—One-Way ANOVA with Tukey post-hoc test. (TIF) [file ppat.1012529.s003.TIF]

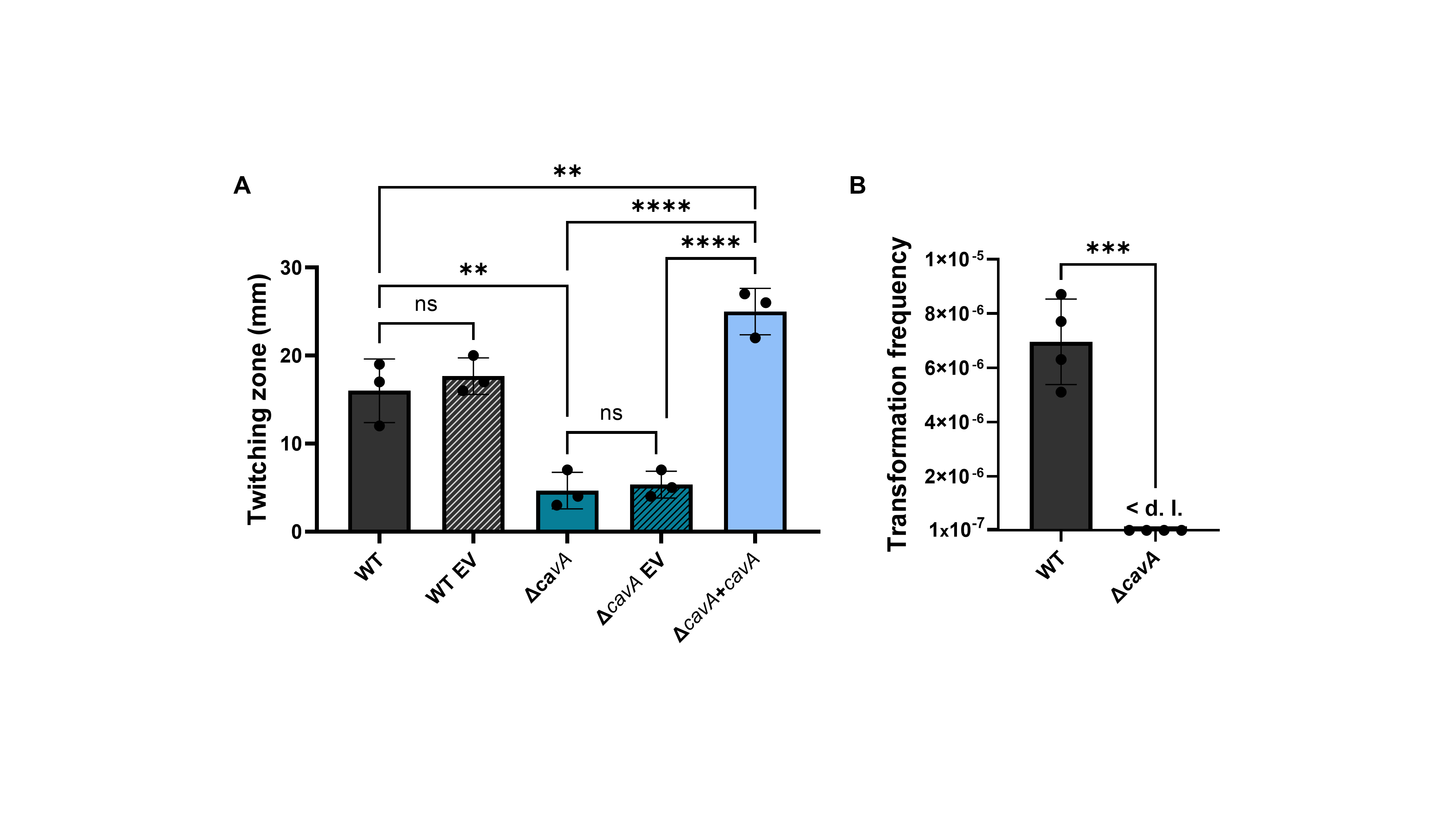

Supplement: S4 Fig — A–Twitching motility of ΔcavA related strains showing that miniTn7 EV has no impact on A. baumannii motility. B–Natural transformation of ΔcavA mutant was bellow the detection limit (< d. l.) and was significantly decreased compared to the WT AB5075. ns p>0.05, ** p<0.01, ***p<0.001, **** p<0.0001—One-Way ANOVA with Tukey post-hoc test (A) and Unpaired t-test (B). (TIF) [file ppat.1012529.s004.TIF]

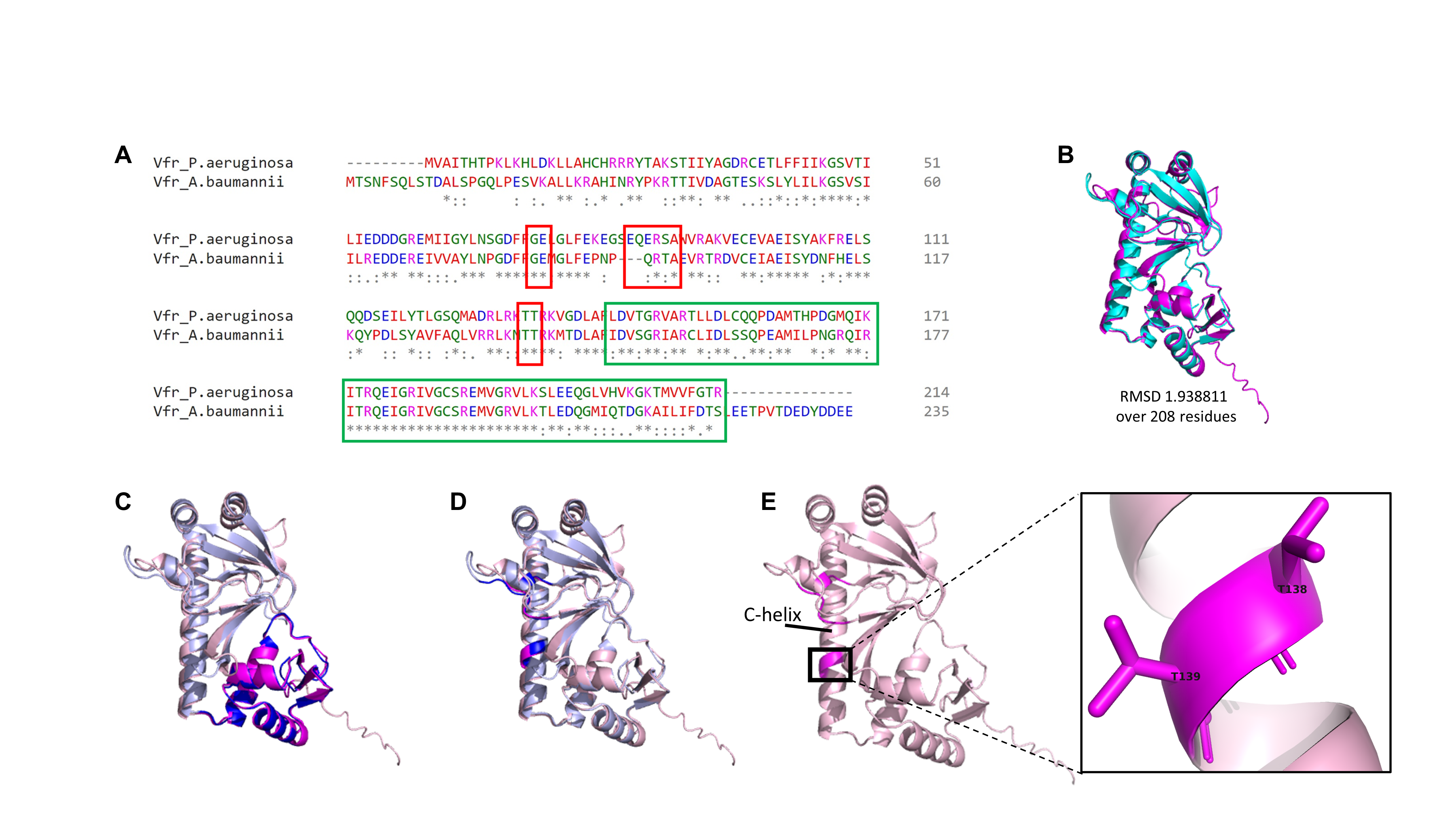

Supplement: S5 Fig — A—Protein sequence alignment comparing Vfr from A. baumannii AB5075 and its orthologue from P. aeruginosa PAO1. Residues involved in the interaction with cAMP, according to Beatson et al. [76], are framed in red. The strong conservation between these residues in both orthologues suggests that both proteins will interact with cAMP with the same specificity. The DNA binding domain of both proteins, as predicted by an InterPro sequence scan (PF00325), appears framed in green. The conservation in this region suggests a similar target promoter sequence for both orthologues. (*): identical residues; (:): highly similar residues; (.): somewhat similar residues. The sequence alignment was performed using Clustal Omega. B-D–Structural alignment of VfrAB5075 and VfrPAO1 in PyMOL. Overall alignment of the two proteins (VfrAB5075 in magenta and VfrPAO1 in cyan) indicates root mean square deviation (RMSD) of 1.938811 over 208 residues (B). Highlighted are the DNA binding domains (C) and cAMP binding sites (D) in VfrAB5075 (magenta) and VfrPAO1 (blue). E–Two residues (threonine 138 and threonine 139) from the VfrAB5075 cAMP binding site (magenta) were modified to alanine and tryptophan respectively resulting in VfrT138A,T139W variant which was subsequently used to demonstrate the necessity of cAMP binding for proper Vfr function in A. baumannii. Protein structure visualised using PyMOL. (TIF) [file ppat.1012529.s005.TIF]

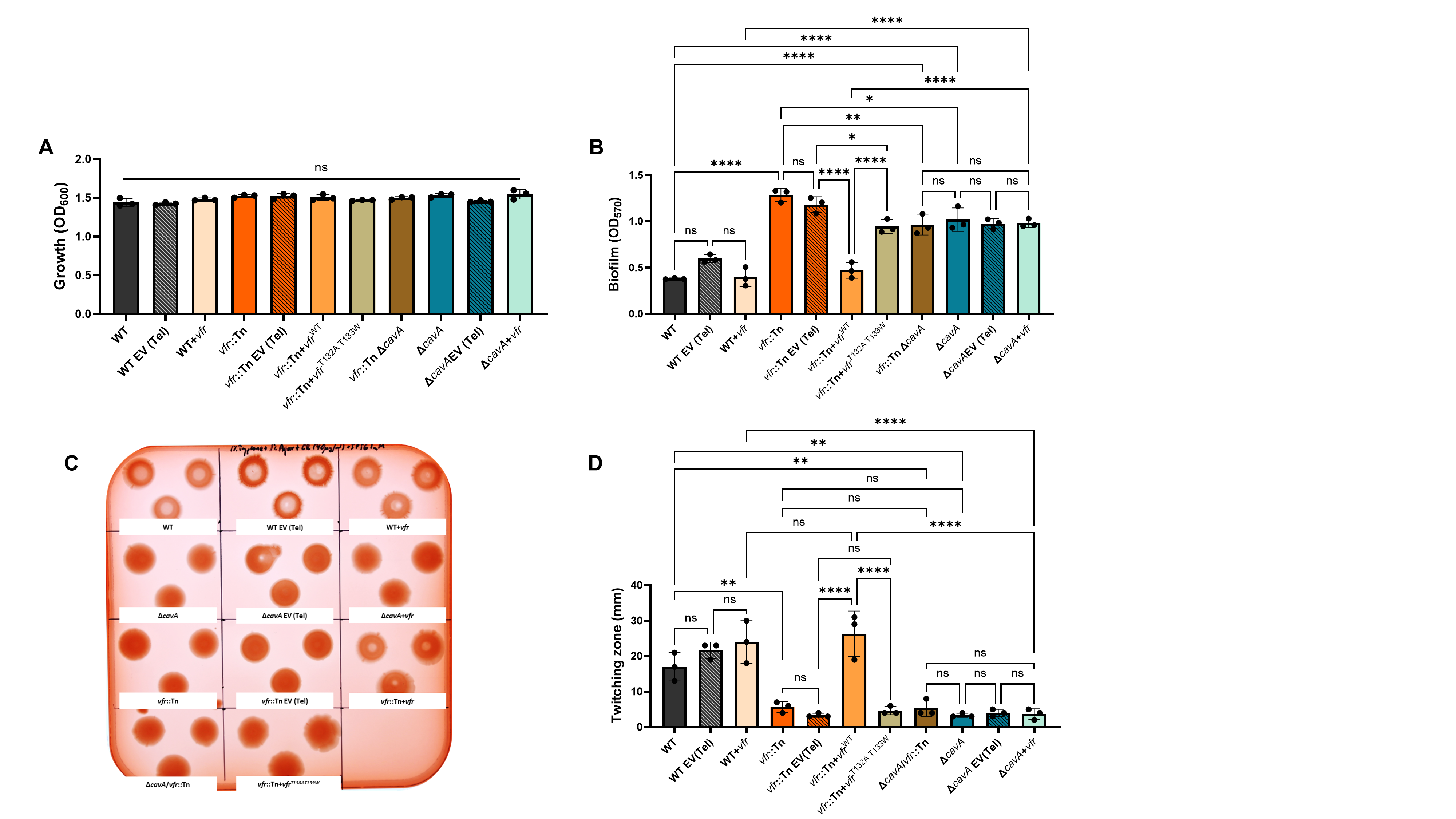

Supplement: S6 Fig — Growth (A), biofilm formation (B), EPS production (C) and motility (D) of cavA and vfr related strains demonstrating that the observed phenotypes were not attributed to growth alternations of the strains. Moreover, the empty miniTn7 system (EV) with Tetracycline (Tc) or Tellurate (Tel) resistance markers, used for the complementations and genes expression in different backgrounds, did not have an effect on any of the tested phenotypes. Growth and biofilm formation were assessed after 24 h at 37°C shaking. EPS production was assessed on Congo agar after 5 days incubation of the strains at 37°C and motility was tested after 48 h on soft agar at 37°C. ns p>0.05, *p<0.05, ** p<0.01, *** p<0.001, **** p<0.0001—One-Way ANOVA with Tukey post-hoc test. (TIF) [file ppat.1012529.s006.TIF]

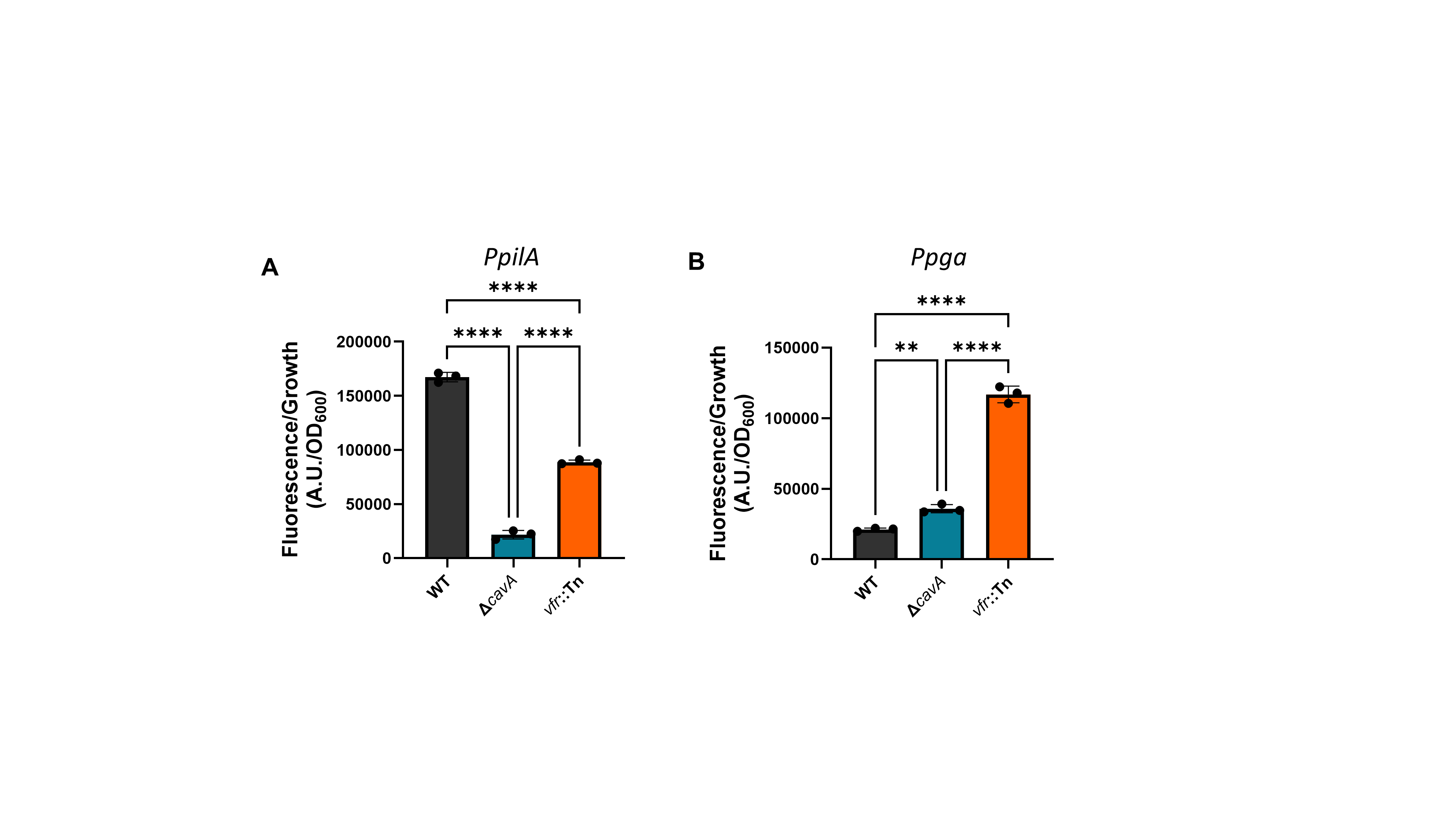

Supplement: S7 Fig — Expression of pilA gene (A) and pga operon (B) is regulated by CavA and Vfr. Promoter regions of pilA and pga fused with gfpmut3 fluorescent reporter (PpilA::gfpmut3 and Ppga::gfpmut3 respectively) were used to assess the effect of CavA and Vfr on their expression. Bacterial cells were harvested from diluted bacterial cultures grown for 4.5 h, after which were resuspended in sterile PBS. Fluorescence (A.U.) at 470-15/515-20 nm excitation/emission was measured to determine the expression of Gfp and thus the expression of each promoter. Optical density at 600 nm (OD600) was measured to determine growth. Data represents the average of three independent repeats ± SD. **p<0.01, ****p<0.0001 –One-Way ANOVA with Tukey post-hoc test. (TIF) [file ppat.1012529.s007.TIF]

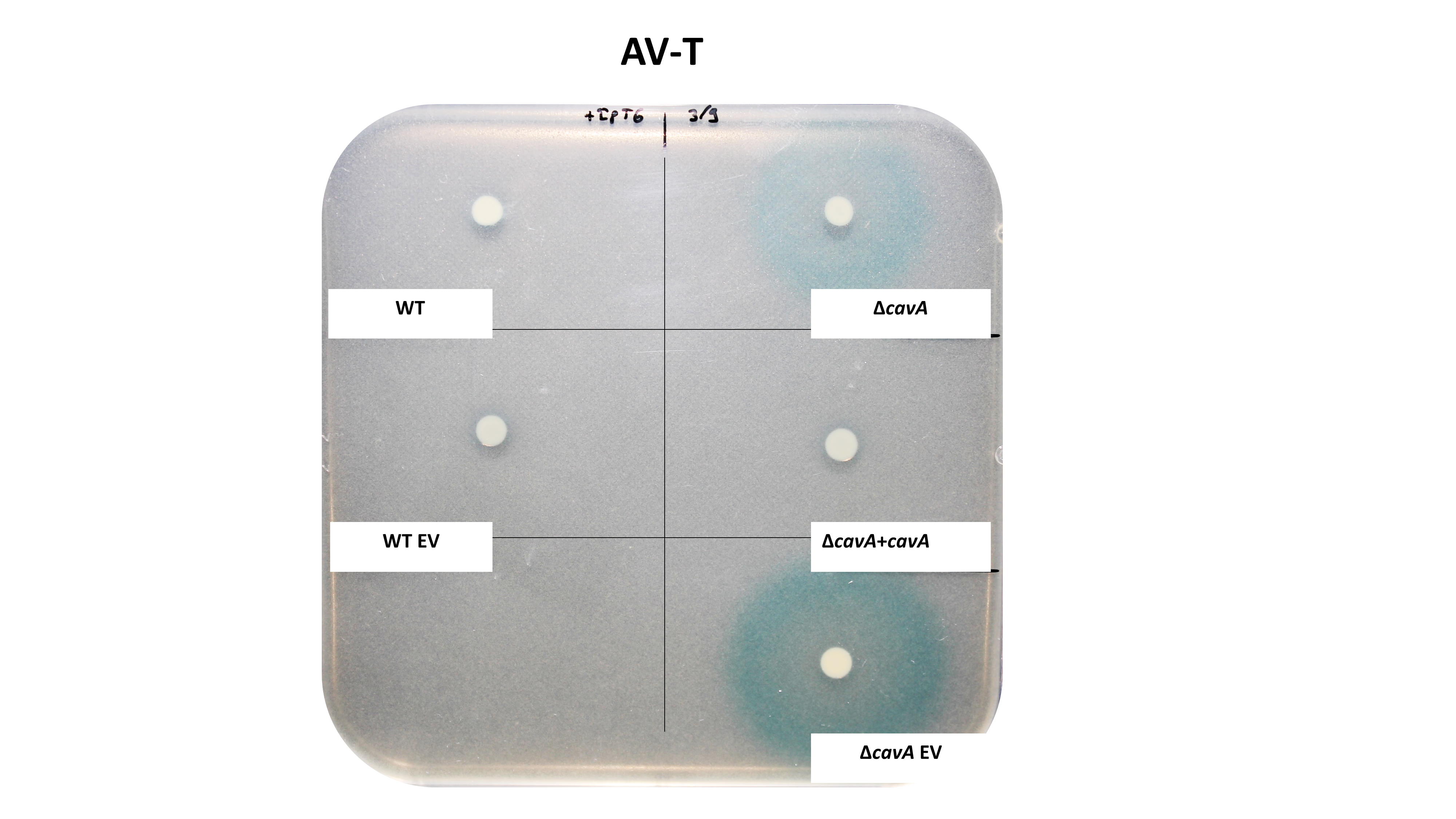

Supplement: S8 Fig — AHL production (indicated by the presence of a blue halo around the colonies) by the AV-T variants of the wild-type (WT) AB5075 and its cavA related derivatives. Dramatic difference was observed in the AHL synthesis, as the deleted ΔcavA mutant had increased AHL production compared to the WT and complemented ΔcavA+cavA strains where AHL secretion was abolished. AHL production was unaffected by the chromosomal insertion of the empty miniTn7 vector (EV) in the WT and ΔcavA backgrounds. (TIF) [file ppat.1012529.s008.TIF]

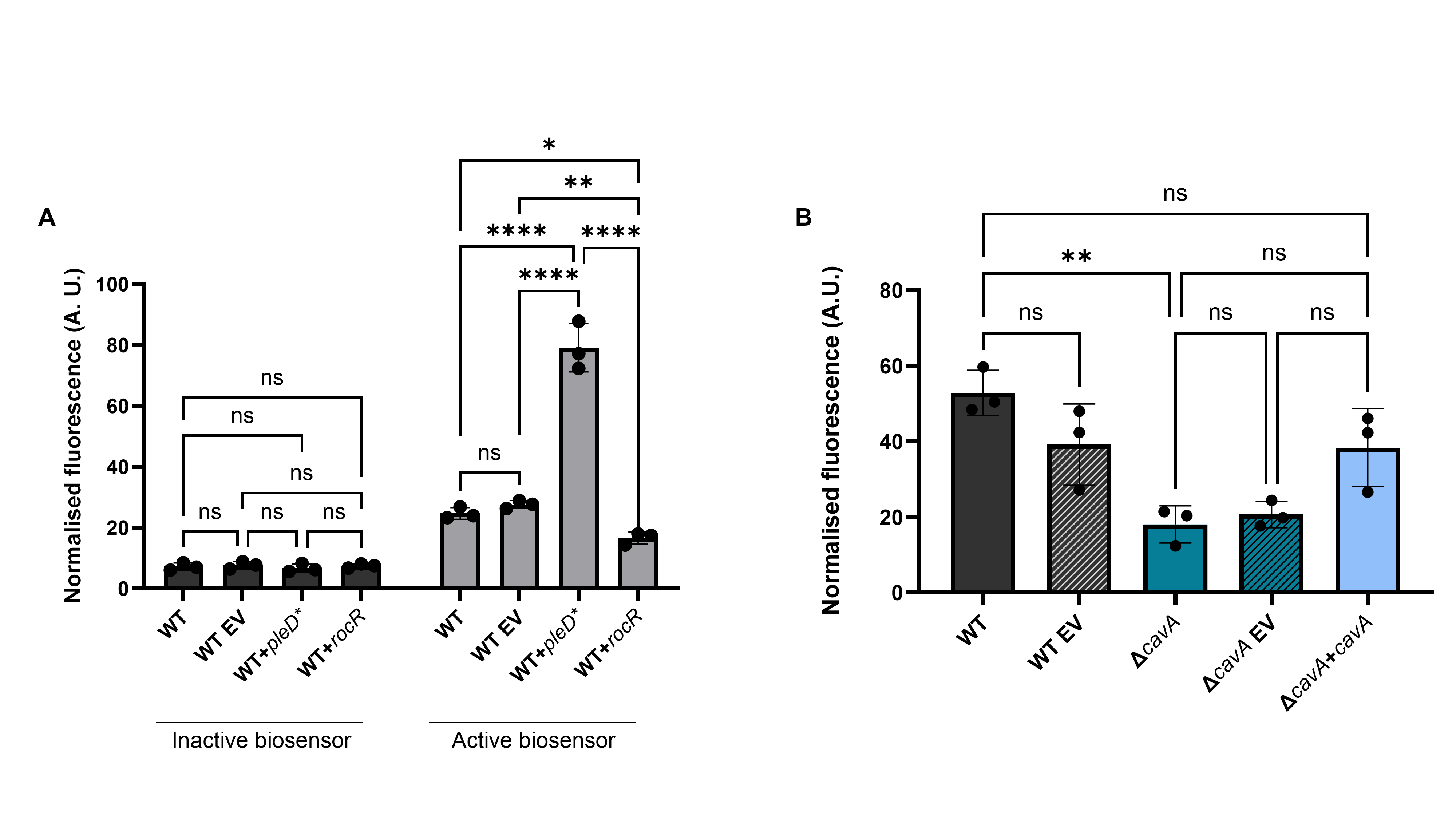

Supplement: S9 Fig — A—Cyclic di-GMP levels in WT AB5075, WT overexpressing constitutively active DGC gene pleD* (WT+pleD*) and WT with empty miniTn7 used for the strain construction. This demonstrates the activity of the modified CensYBL-Ab in detecting the elevated c-di-GMP levels in the WT+pleD* strain. The inactive CensYBL*-Ab biosensor was used to demonstrate that the increase in the signal was due to the changing c-di-GMP levels. B–Cyclic di-GMP levels in cavA related strains compared to the parental WT AB5075. The empty miniTn7 was used as control which demonstrates the empty vector did not have an effect on the c-di-GMP levels in the WT or the deleted ΔcavA mutant. ns p>0.05, ** p<0.01, **** p<0.0001—Two-Way ANOVA (A) and One-Way ANOVA (B) with Tukey post-hoc test. (TIF) [file ppat.1012529.s009.TIF]

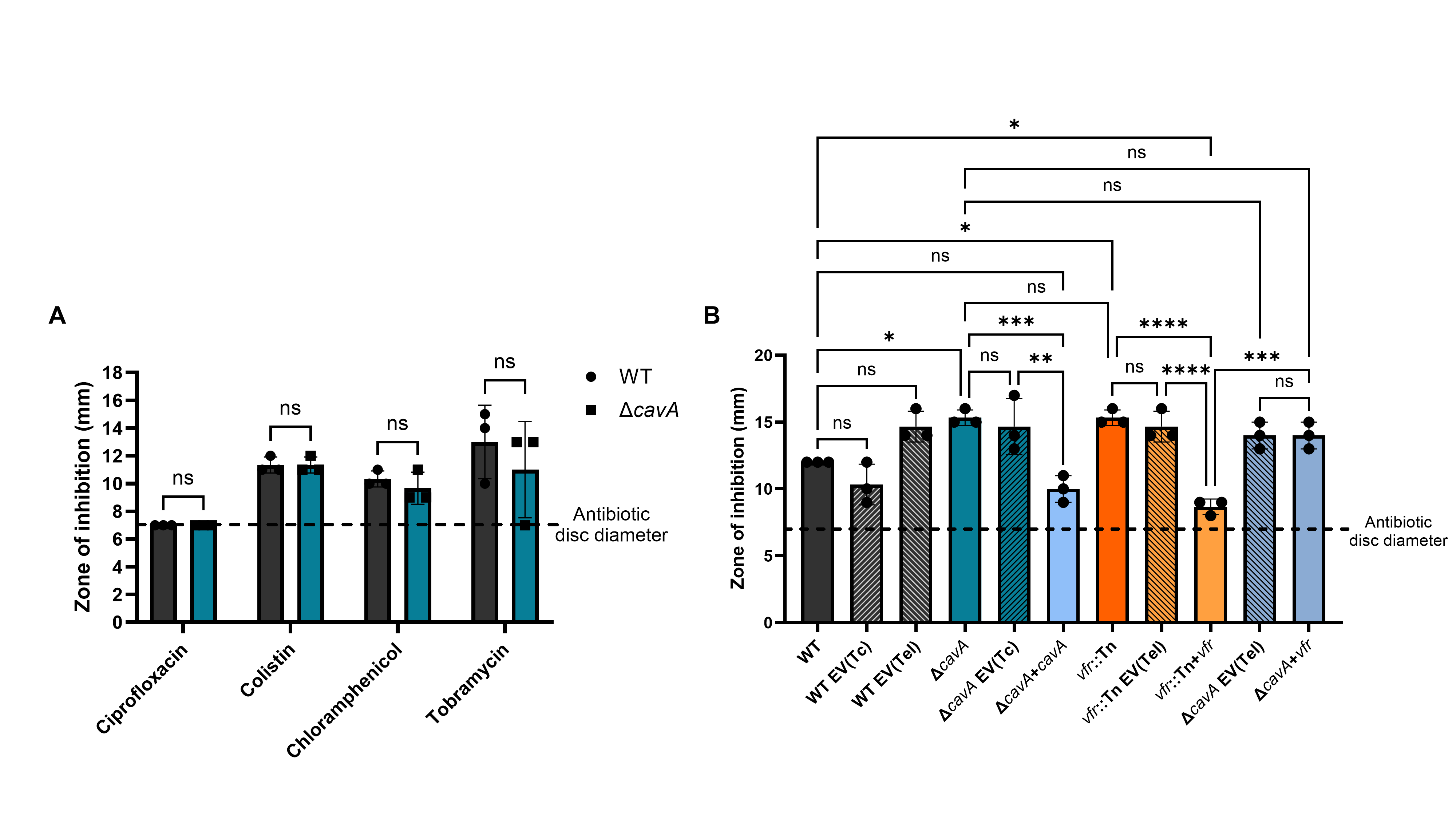

Supplement: S10 Fig — A–WT AB5075 and ΔcavA resistance to different classes of antibiotics such as quinolones (ciprofloxacin, 5 μg), polymyxins (colistin, 10 μg), amphenicols (chloramphenicol, 50 μg) and aminoglycosides (tobramycin, 30 μg). B—Full dataset of WT and its cavA and vfr related derivatives to fosfomycin (50 μg). Strains harbouring empty miniTn7 (EV) with tetracycline (Tc) or tellurate (Tel) resistance cassette were used as controls demonstrating that the resistance phenotype was unaffected by the presence of the empty vectors. ns p>0.05, *p<0.05, ** p<0.01, *** p<0.001, **** p<0.0001 –Two-Way ANOVA with Sidak post-hoc test (A) and One-Way ANOVA with Tukey post-hoc test (B). (TIF) [file ppat.1012529.s010.TIF]
